# Association Between Gut Microbiota and Pneumonia Risk: A Systematic Review and Mendelian Randomization

**DOI:** 10.7150/ijms.114372

**Published:** 2025-07-28

**Authors:** Qingping Deng, Yuanyuan Liu, Hui Rong, Qing Liu, Rongyuan Yang

**Affiliations:** 1 State Key Laboratory of Traditional Chinese Medicine Syndrome, Guangdong Provincial Hospital of Chinese Medicine, The Second Affiliated Hospital of Guangzhou University of Chinese Medicine, 111 Dade Road, Guangzhou, Guangdong 510120, China.; 2 Hubei University of Traditional Chinese Medicine, Wuhan Hospital of Traditional Chinese Medicine, Wuhan 430065, China.

**Keywords:** COVID-19, gut microbiota, meta-analysis, Mendelian randomization

## Abstract

**Background:** The gut-lung axis represents a critical pathway potentially modulating COVID-19 pathogenesis. We employed meta-analysis to investigate the Mendelian randomization (MR) studies for the putative causal relationships between gut microbiota composition/metabolites and COVID-19 severity.

**Methods:** Adhering to PRISMA 2020 guidelines, we conducted a systematic review of MR studies (PubMed/Web of Science/Embase/Scopus/Cochrane; inception to June 2024). Data from 11 studies (aggregating 32,748,274 participants; 1,487 SNPs) underwent meta-analysis across four COVID-19 severity strata including susceptibility, infection, hospitalization, and critical disease. Study quality was evaluated using a validated MR framework assessing 32 core assumptions.

**Results:** Elevated COVID-19 susceptibility risk was associated with *Actinobacteria* (OR 1.16, 95% CI 1.06-1.26) and *Negativicutes* (1.06, 1.03-1.09), whereas protective effects emerged for *Oxalobacter* (0.84, 0.71-0.99) and *Ruminococcaceae UCG014* (0.88, 0.78-0.99). For COVID-19 infection, *Negativicutes* conferred increased risk (1.13, 1.02-1.26), while the *Ruminococcus* torques group (0.54, 0.39-0.74) and *Parasutterella* (0.90, 0.83-0.97) demonstrated protection. Hospitalization risk elevated with *MollicutesRF9* (1.13, 1.04-1.22) and *Alloprevotella* (1.25, 1.07-1.45), contrasting with *butyrate* (0.97, 0.94-0.99) and *Ruminiclostridium*6 (0.81, 0.69-0.94) showing protective associations. Severe COVID-19 risk increased with *Actinobacteria* (1.20, 1.01-1.42), *Bifidobacterium* (2.09, 1.15-3.81), and *Alloprevotella* (1.66, 1.36-2.01), while *Oxalobacter* (0.84, 0.76-0.92) and *Subdoligranulum* (0.82, 0.76-0.89) exhibited protection. Notably, *Actinobacteria*, *Negativicutes,* and *Alloprevotella* constituted consistent risk factors across severity strata, whereas *Oxalobacter* and *Parasutterella* showed trans-stage protective effects. *Butyrate* production specifically attenuated hospitalization risk, and *Bifidobacterium* demonstrated strikingly elevated critical disease risk, contrasting with typical probiotic associations.

**Conclusions:** This meta-analysis of MR studies provides robust evidence for severity-specific causal effects of the gut microbiota on COVID-19 outcomes. The identified microbial taxa and metabolites provide potential biomarkers for clinical risk stratification and targets for novel adjuvant therapeutic strategies.

## Introduction

Human coronaviruses periodically emerge as significant global health threats. The most recent and impactful example is severe acute respiratory syndrome coronavirus 2 (SARS-CoV-2), the causative agent of COVID-19. Global surveillance data indicate a substantial disease burden: as of July 7, 2024, worldwide cumulative confirmed SARS-CoV-2 infections exceeded 775 million cases, with reported fatalities surpassing 7.05 million 1. The United States (103,436,829 cases), China (99,365,162 cases), and India (45,040,752 cases) reported the highest national cumulative caseloads 1. While the exponential growth observed during initial pandemic peaks has plateaued, transmission rates remain persistently elevated 2-4. The pandemic has generated profound health, economic, and societal consequences globally. Its prolonged duration and extensive spread underscore the critical need for continued investigation into its multifaceted health and socioeconomic impacts.

The gastrointestinal tract constitutes the body's largest immune organ, where resident microbiota critically modulate host immunity and nutritional metabolism 5. Dominated by Firmicutes, Bacteroidetes, Actinobacteria, Fusobacteria, Proteobacteria, Verrucomicrobia, and Cyanobacteria 6, this microbial consortium co-evolves with the host to provide pathogen defense. Clinical evidence links specific gut bacterial populations to pneumonia severity 7-10. COVID-19 patients exhibit significant depletion of anti-inflammatory butyrate-producing bacteria during acute infection compared to healthy controls 9, 10 SARS-CoV-2 invasion via respiratory epithelium damages mucosal barriers in both pulmonary and gastrointestinal systems. This compromise facilitates viral dissemination and secondary bacterial infections, inducing intestinal dysbiosis. Characteristic alterations include reduced commensal bacteria abundance with concurrent expansion of opportunistic pathogens, fundamentally disrupting gut ecological homeostasis 11.

Mendelian Randomization (MR) represents a robust method for investigating causal relationships between variables. This approach employs genetic variants as instrumental variables, assigning individuals to exposure groups based on naturally occurring genetic differences. By leveraging Mendel's second law of inheritance, MR minimizes confounding and reverse causation biases that limit conventional observational studies. The method consequently enables rigorous causal inference in complex biological systems.

Current literature on COVID-19 and gut microbiota includes numerous reviews, though many provide limited mechanistic analysis of intestinal microorganisms' involvement 12-14. While Mendelian randomization (MR) studies have investigated gut microbiota-COVID-19 relationships 15, a comprehensive synthesis of this evidence remains unavailable. This study addresses this gap through meta-analysis of existing MR investigations on COVID-19-gut microbiota associations. We further stratify analyses by COVID-19 severity to establish an evidence-synthesis framework for disease-microbiota interactions.

## Methods

### Study design

This study followed the Preferred Reporting Items for Systematic Evaluation and Meta-Analysis Protocols (PRISMA) 2020 guidelines. The study protocol is registered with PROSPERO under the registration number CRD42024570240.

### Literature search strategies

This Mendelian randomization (MR) study investigated the causal effects of gut microbiota and metabolites on pneumonia risk. We systematically searched PubMed, Web of Science, Embase, Scopus, and the Cochrane Library for English-language publications published from database inception until June 25, 2024. The search strategy combined Medical Subject Headings (MeSH) and free-text terms (e.g., "Mendelian randomization","COVID-19","covid-19 virus disease","2019-ncov diseases","gut microbiota","microflora intestinal","gut microbiota metabolites"). Studies on other pneumonias identified under these search terms were also included; the full search strategy is detailed in **Supplementary Table S1**. **Supplementary Table S1** is used to present the search strategies and results in each database.

### Study selection

Literature records were imported into NoteExpress. Two reviewers independently screened titles, abstracts, and full-text articles against uniform eligibility criteria. Disagreements were resolved through consensus with a third reviewer.

### Quality assessment

The quality evaluation of this article was conducted using the methodological framework for MR studies developed by Mengyuan Wang *et al.* 16. This framework comprises six key components: the completeness of instrumental variable analysis, validation of the assumptions of association, independence, and exclusivity, implementation of sensitivity analyses, consideration of population stratification, and examination of nonlinear associations. The criteria for assessing each component are detailed in **Table 1**. By systematically applying these principles, we ensured a comprehensive and rigorous evaluation of the article's methodological quality.

MR studies necessitate full IV analysis to ensure quality **(Table 1A)**. Simultaneous fulfillment of the 3 core assumptions of association (genetic variants are associated with the exposure phenotype), independence (genetic variants are independent of confounders affecting the association), and exclusivity (genetic variants affect the outcome only through exposure) is necessary for reliable results. Single-sample and two-sample MR studies often rely on different methods to test the hypothesis of association of genetic variants with exposure phenotypes **(Table 1 B)**. Multiplicity of effects of genetic variation is prevalent, and the independence and exclusivity hypotheses may be violated if genetic tools influence outcomes through factors other than the exposure of interest, and the two hypotheses can generally be tested together **(Table 1 C)**.MR studies assessed the robustness of the results through sensitivity analyses **(Table 1 D)**. Given the heterogeneity of genetic susceptibility across races, attention also needs to be paid to the potential impact of population stratification **(Table 1 E)**. In addition, we were concerned about how well studies explored and rated potential nonlinear associations between exposure and outcomes **(Table 1 F)**. Given that there is no MR methodology that uses summary statistics to explore nonlinear associations, and that most MR studies focus only on linear associations between exposures and outcomes, we judged studies with "good" ratings on all five items as high-quality MR studies, relying primarily on entries A to E in **Table 1**.

### Data extraction

Two investigators independently extracted data including: title, authors, publication year, disease phenotype, case/control counts, microbiota/metabolite features, SNP numbers, ORs (95% CIs), and IVW causal estimates. Discrepancies were resolved through iterative discussion. Three researchers implemented this process: two performed literature review and data extraction, with the third overseeing result verification and facilitating consensus discussions when required.

### Statistical analysis

Study data were standardized prior to analysis to ensure methodological consistency. Using Review Manager (RevMan v5.4, Cochrane Collaboration), we conducted: (1) risk-of-bias assessments, (2) data harmonization, and (3) meta-analyses. Pooled odds ratios (ORs) with 95% confidence intervals (CIs) quantified associations between gut microbiota metabolites and pneumonia. Heterogeneity was evaluated using Cochran's Q-test and I^2^ statistics, with Cochrane-recommended significance thresholds (PQ < 0.10 or I^2^ > 50%). Random-effects models were applied when significant heterogeneity was detected. Publication bias was assessed through funnel plot symmetry examination supplemented by Egger's regression tests. Sensitivity analyses evaluated the robustness of findings. For exposures in studies reporting F-statistics (9 out of 11) 17-25, all F-statistics exceeded 10, meeting the relevance assumption requirement for instrument strength; F-statistics were not reported in the other two studies 26, 27.

## Results

### Literature search results and study characteristics

**Figure 1:**
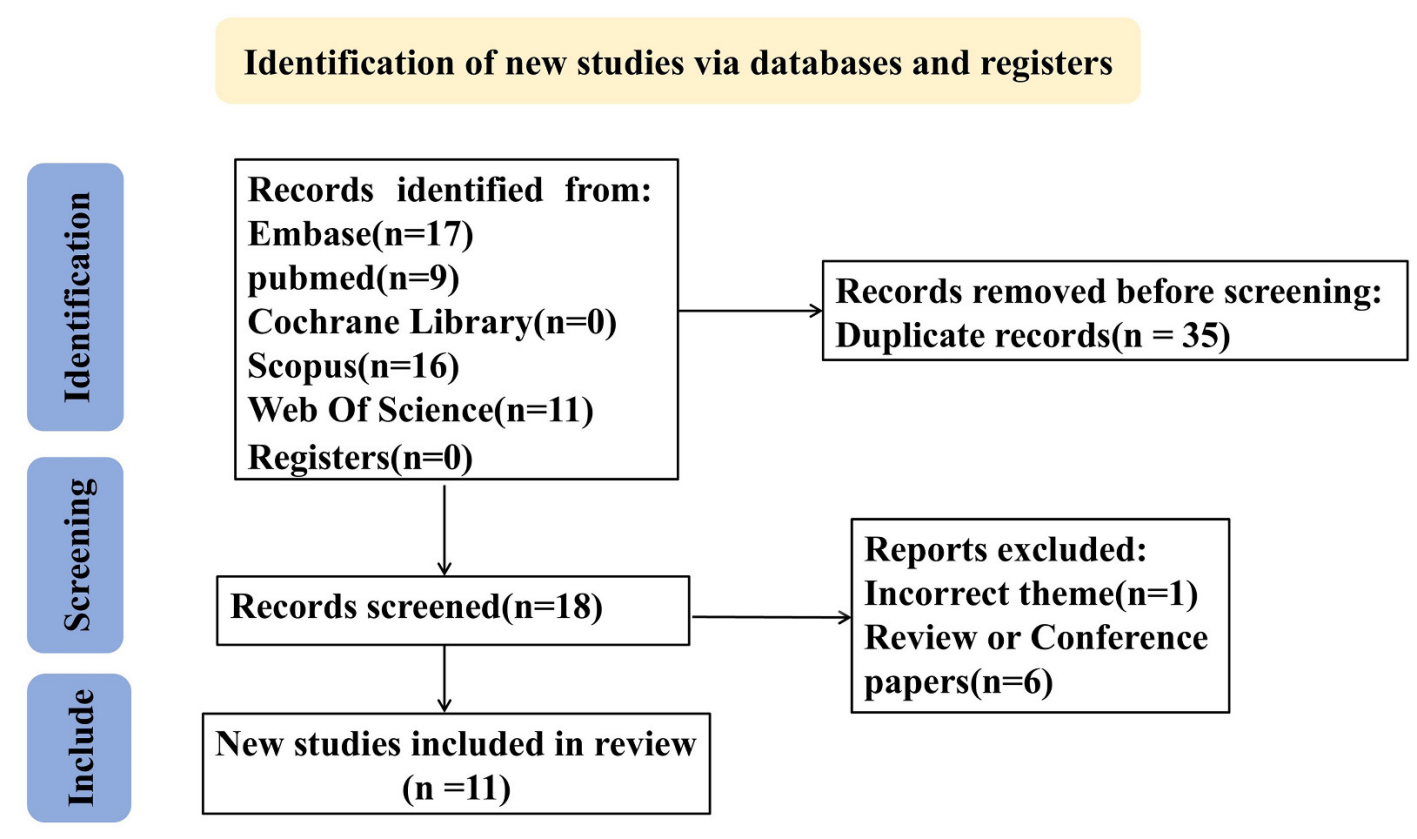
** Flow diagram of the data collection and analysis in this study**.

Retrieved records were imported into NoteExpress (Aegean Software, China) for standardized management. The search strategy combined Medical Subject Headings (MeSH) and free-text terms including "Mendelian randomization","COVID-19","covid-19 virus disease", "2019-ncov diseases", "gut microbiota", "microflora intestinal" and "gut microbiota metabolites" with intentional inclusion of pneumted onia-reladisorders through COVID-19 terminology (See **Supplementary Table S1** for search strategies and results in each database). During screening, 34 publications were excluded based on: (1) cross-database duplication, (2) supplementary materials, (3) funding announcements, (4) review articles/meta-analyses, or (5) non-pneumonia relevance. Two investigators (YYL and QPD) independently performed quality assessment and data extraction, with discordant evaluations resolved by third-reviewer (QL) arbitration. The final analysis incorporated 11 eligible studies 17-27, encompassing 32,748,274 participants and 1,487 single-nucleotide polymorphisms (SNPs). Study characteristics are summarized in **Table 2**, while the screening process is depicted in the PRISMA flowchart **(Figure 1)**.

### Quality assessment

**Figure 2:**
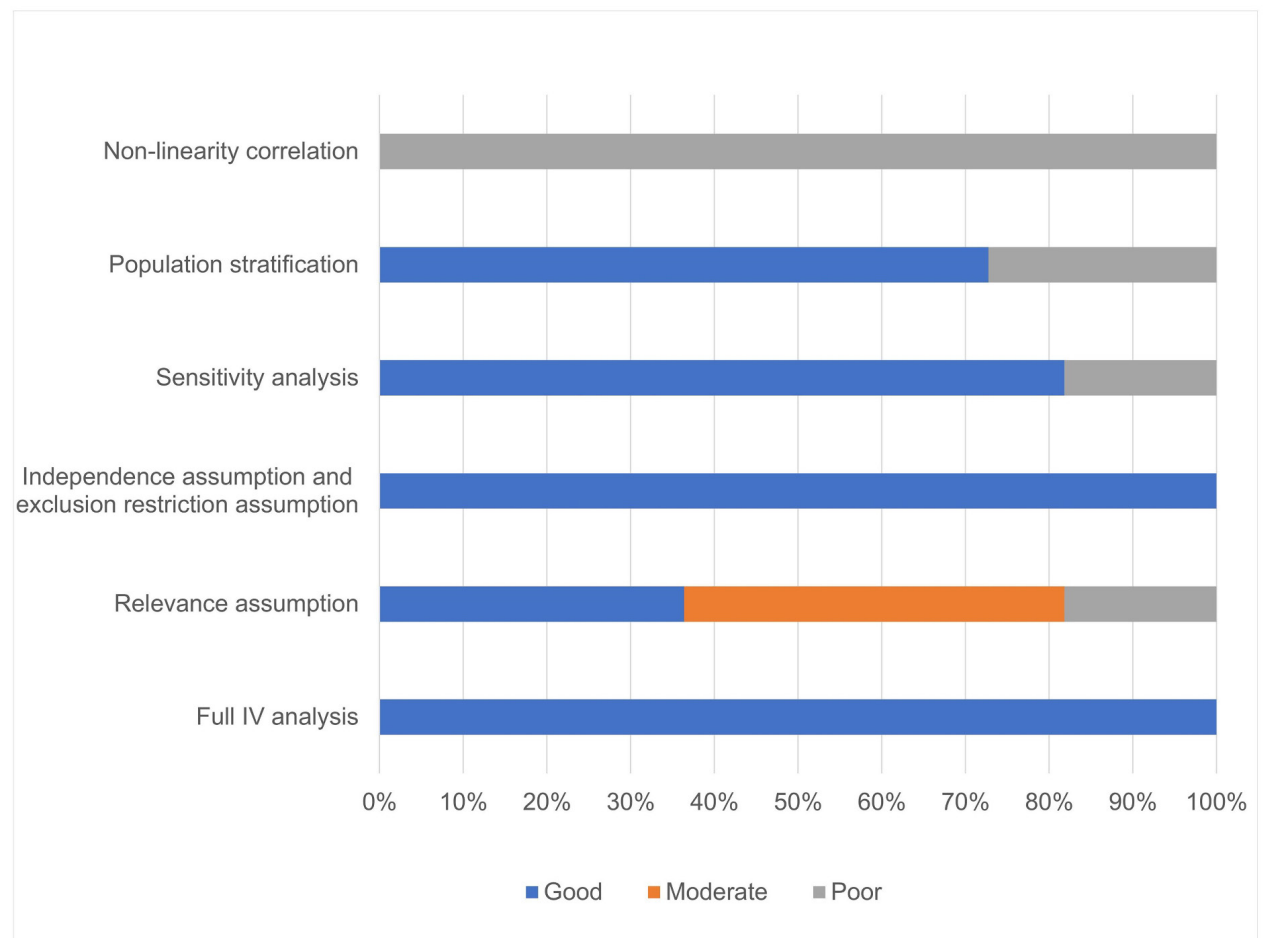
** Quality evaluation results of a Mendelian randomized study on the relationship between pneumonia and intestinal microbiota and its metabolites**.

Our meta-analysis of 52 gut microbiota taxa identified consistent microbial signatures associated with pneumonia severity **(Supplementary Table S2** provides a comprehensive summary of the effects of different gut microbiota taxa on COVID-19 pneumonia.), including COVID-19 outcomes. Among the 11 studies, all performed a complete IV analysis, 9 validated the three core hypotheses of MR research, 9 conducted sensitivity analyses, and 8 showed no evidence of population stratification. Ultimately, 4 studies were deemed high-quality MR research, as illustrated in **Figure 2**.

### Association between key microbial taxa and pneumonia severity

**Figure 3:**
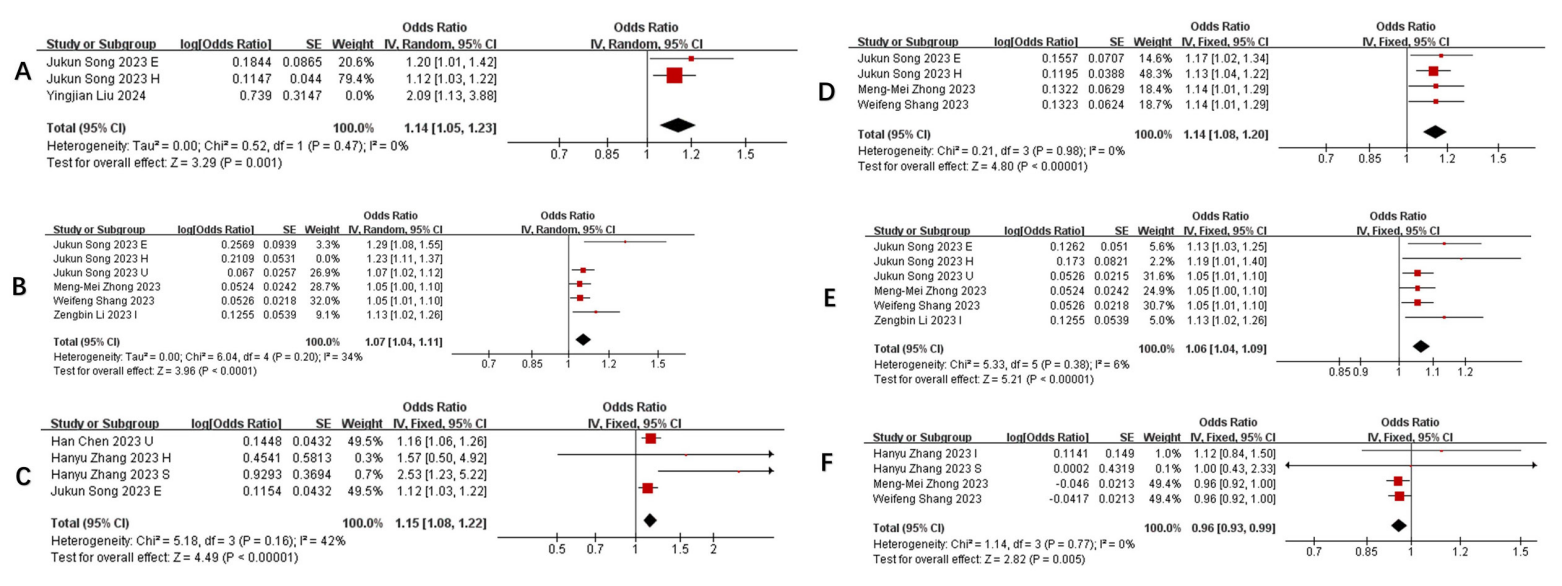

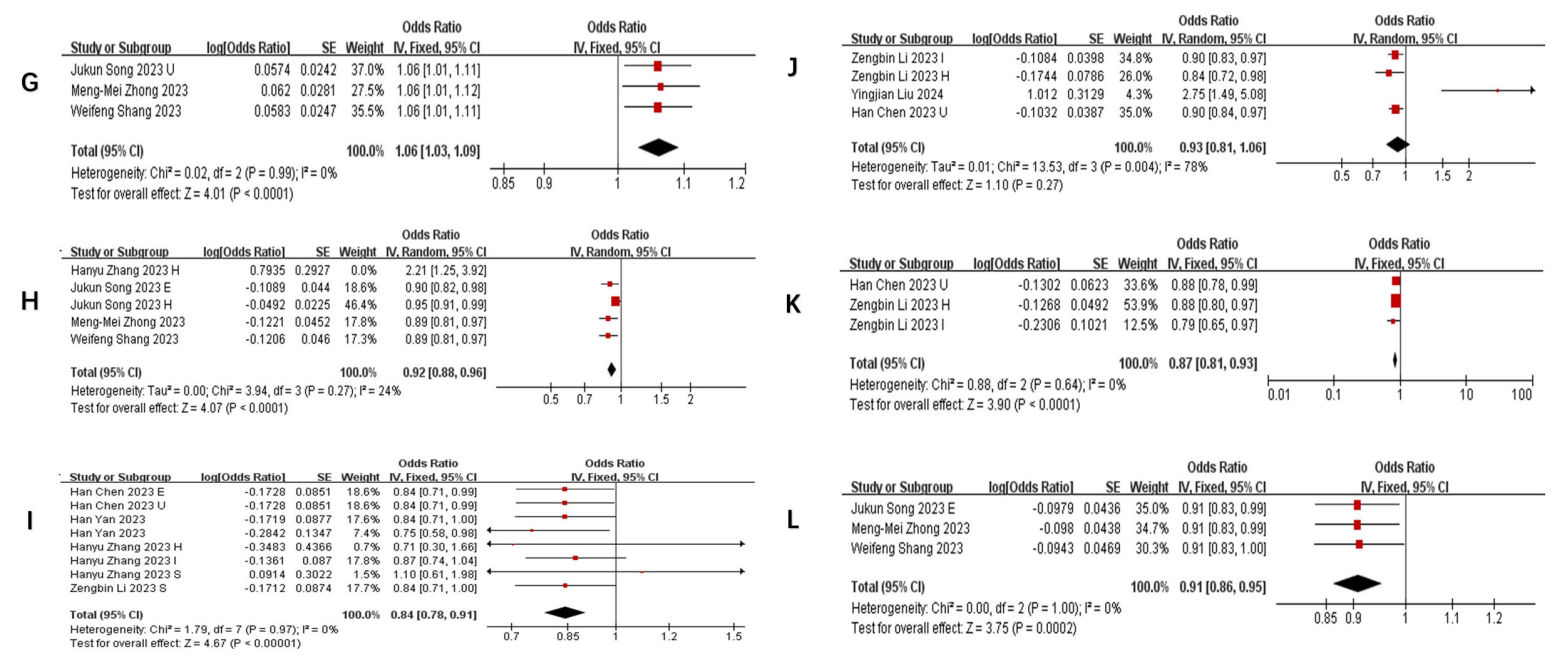

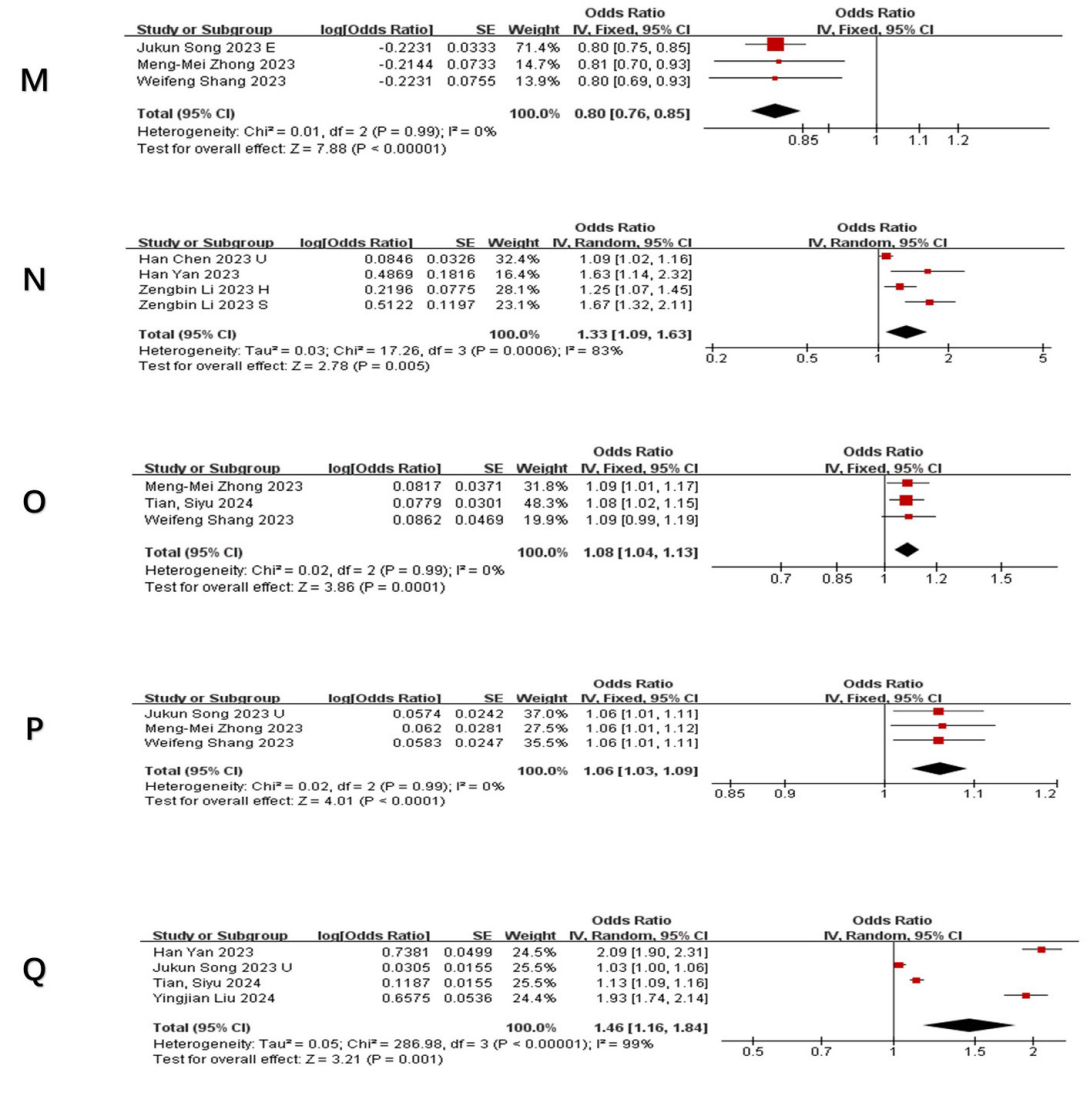
** Forest plot of influence of different gut microbiota on the severity of pneumonia. A)** Relationship between phylum actinobacteria.id and pneumonia; **B)** Relationship between class Negativicutes and pneumonia; **C)** Relationship between class Actinobacteria and pneumonia; **D)** Relationship between order MollicutesRF9 and pneumonia;** E)** Relationship between order Selenomonadales and pneumonia; **F)** Relationship between family Streptococcaceae and pneumonia; **G)** Relationship between genus Bacteroides and pneumonia; **H)** Relationship between genus Tyzzerella3 and pneumonia; **I)** Relationship between genus Oxalobacter and pneumonia; **J)** Relationship between genus parasutterella and pneumonia; **K)** Relationship between genus Ruminococcaceae UCG014 and pneumonia; **L)** Relationship between RuminococcaceaeUCG011 and pneumonia; **M)** Relationship between genus Subdoligranulum and pneumonia; **N)** Relationship between genus Alloprevotella and pneumonia; **O)** Relationship between genus RikenellaceaeRC9 and pneumonia; **P)** Relationship between genus Bacteroides and pneumonia; **Q)** Relationship between genus Bifidobacterium and pneumonia.

**Table 3** summarizes taxa with the strongest and most consistent associations (P<0.01) across ≥3 studies (The complete Summary of Bacterial Flora in COVID-19 Patients with Different Severities is presented in **Supplementary Table S3**). Notably: Positive associations: *Phylum Actinobacteria.id* **(Figure 3 A)***, Class Negativicutes **(*****Figure 3 B)***, Class Actinobacteria* **(Figure 3 C)***, Order MollicutesRF9* **(Figure 3 D)***, Order Selenomonadales* **(Figure 3 E)***, Family Bacteroidaceae* **(Figure 3 G)***, Genus Alloprevotella* **(Figure 3 N)***, Genus RikenellaceaeRC9* **(Figure 3 O)***, Genus Bifidobacterium* **(Figure 3 Q)** and *Genus Bacteroides* **(Figure 3 P)** were repeatedly linked to increased pneumonia severity*.* Negative associations: *Family Streptococcaceae* **(Figure 3 F)***, Genus Tyzzerella3* **(Figure 3 H)***, Oxalobacter* **(Figure 3 I)***, Parasutterella* **(Figure 3 J)***, RuminococcaceaeUCG014* **(Figure 3 K)***, RuminococcaceaeUCG011* **(Figure 3 L)***,* and *Subdoligranulum* **(Figure 3 M)** showed protective effects. Heterogeneity adjustments (e.g., exclusion of outlier studies (19, 23, 27) strengthened these associations (I² reduced to 0-34%).

### Gut microbiota dynamics across COVID-19 severity stages

**Figure 4:**
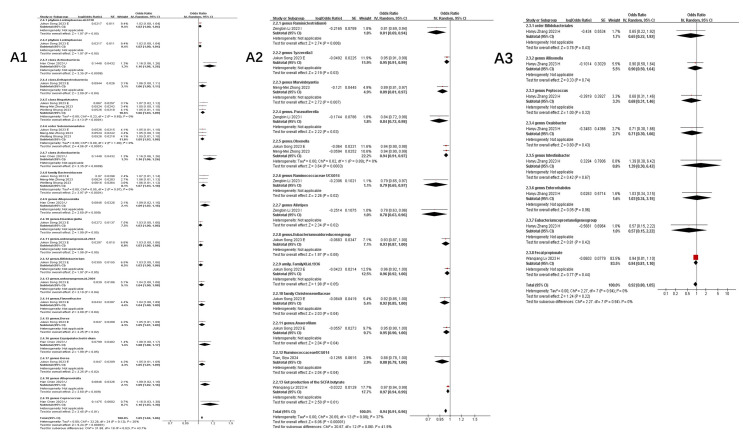

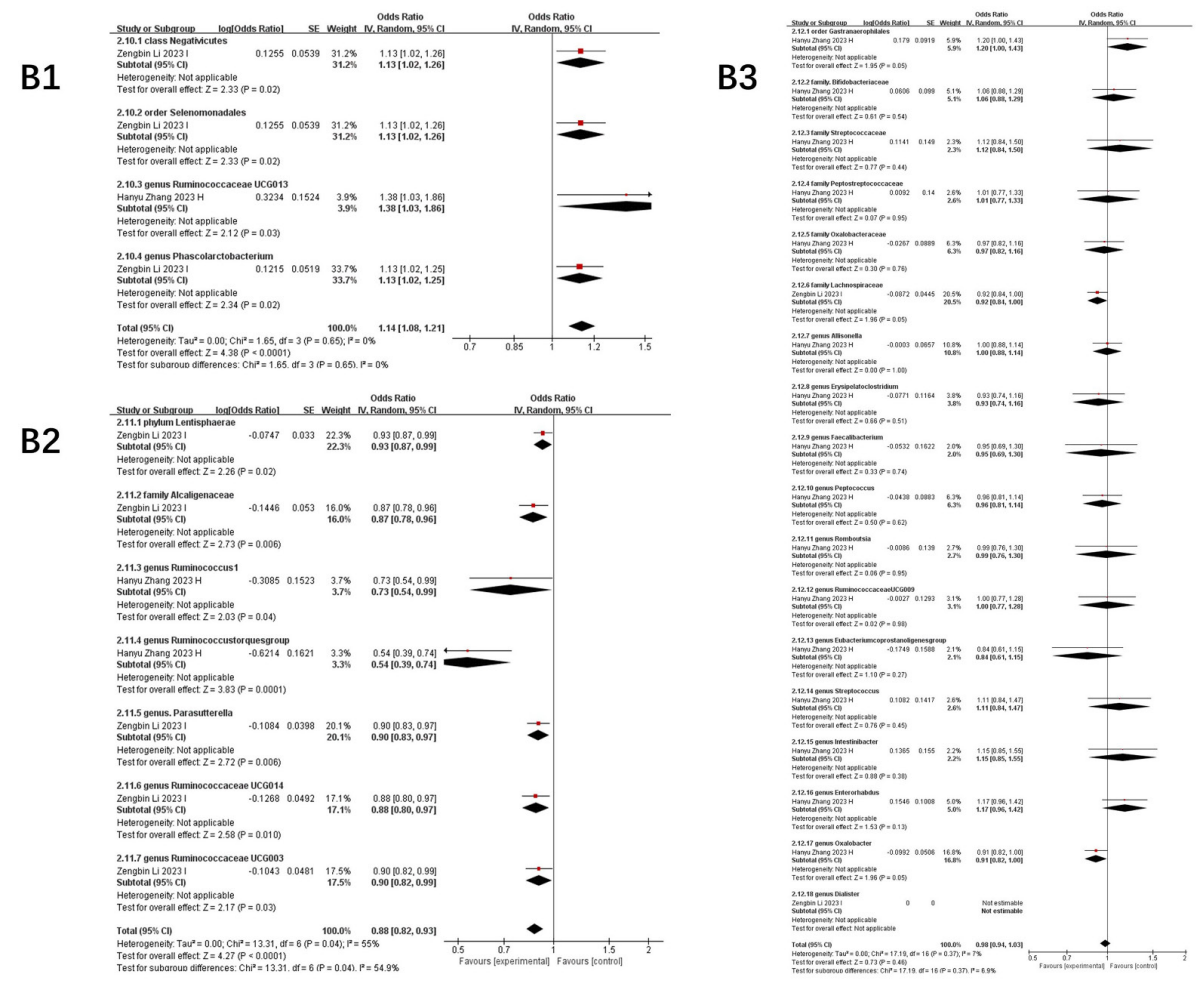

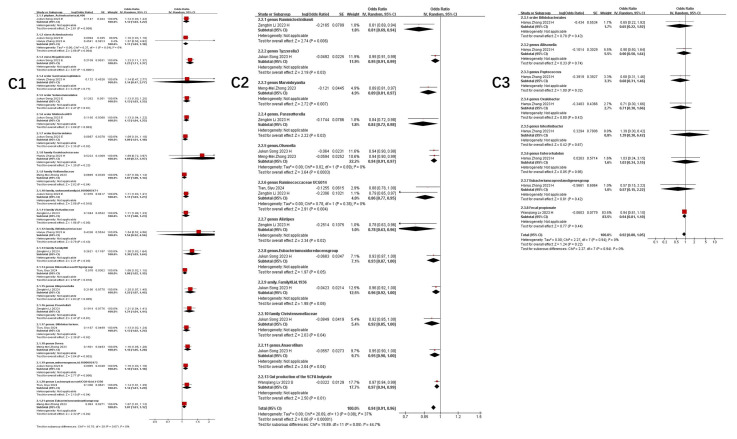

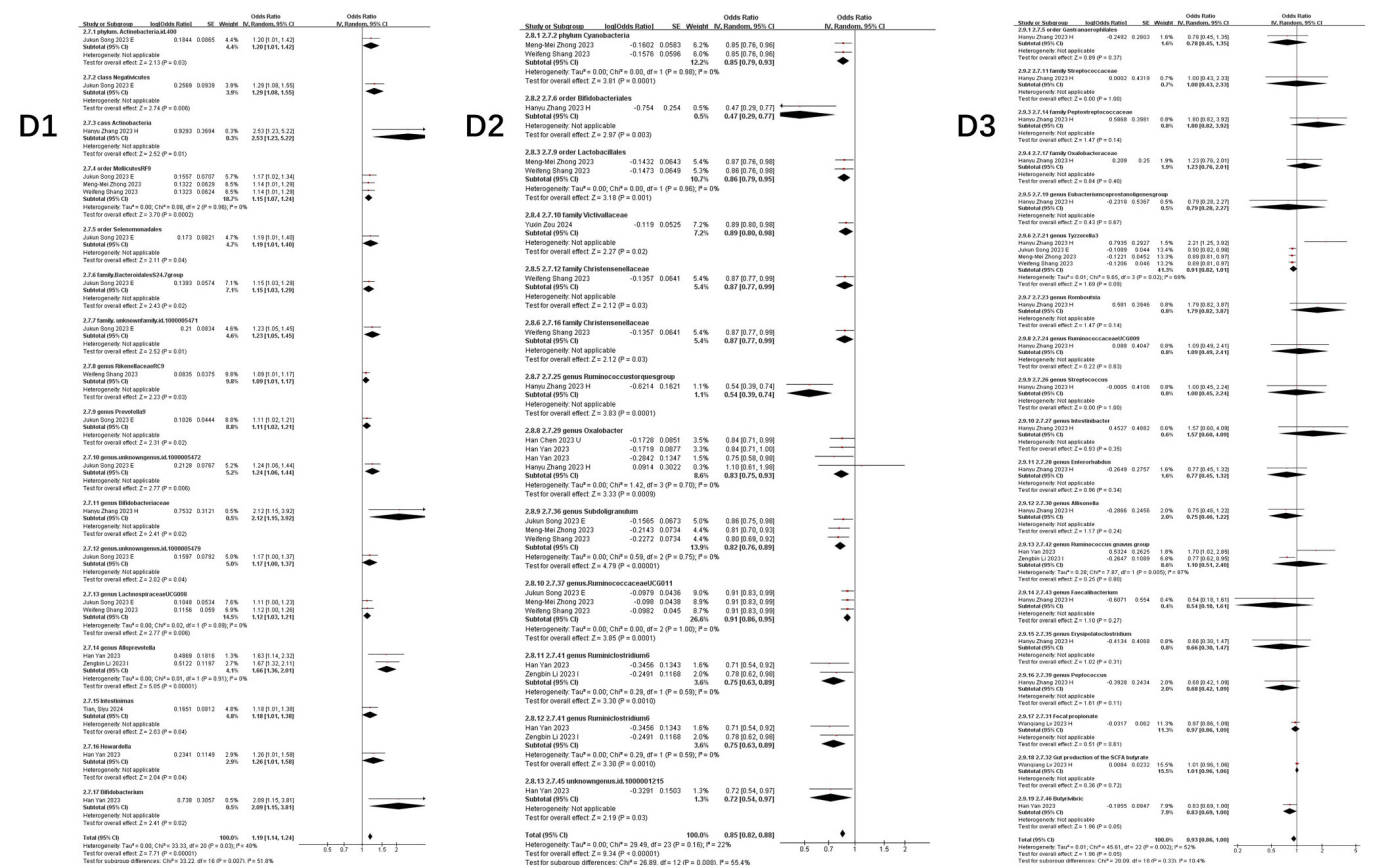
** Forest plot of summary of gut microbiota of different COVID-19 severities. A)** COVID-19 susceptibility; **B)** COVID-19 infection; **C)** COVID-19 hospitalization; **D)** COVID-19 severe.

Figure 4 illustrates taxa significantly associated (P<0.05) with COVID-19 susceptibility, infection, hospitalization, and severe disease. The primary analysis of this study focused on the association between the gut microbiome and COVID-19 risk. Secondary, exploratory analyses of other outcomes (e.g., BP, BLA) are presented in Supplementary Table S3.

### COVID-19 susceptibility

Key microbial signatures identified through Mendelian randomization analysis **(Figure 4 A):**

Risk-enhancing taxa: *genus Dorea* (1.05 [1.01-1.09]), *class Actinobacteria* (1.16 [1.06-1.26]), *class Negativicutes* (1.06 [1.03-1.09]), *phylum Lentisphaerae* (1.02 [1.00, 1.04]), *genus Alloprevotella* (1.09 [1.02, 1.16]), *order Selenomonadales* (1.05 [1.03, 1.08]), *family Bacteroidaceae* (1.07 [1.03, 1.10]), *genus Coprococcus* (1.16 [1.03, 1.30]), and others **(Figure 4 A1)**.

Protective taxa: *genus Oxalobacter* (0.84 [0.71-0.99]), *genus Ruminococcaceae UCG014* (0.88 [0.78-0.99]), *genus Parasutterella* (0.90 [0.84, 0.97]), *class Gammaproteobacteria* (0.94 [0.91, 0.97]), and *family Streptococcaceae* (0.96 [0.93, 0.99]), among others **(Figure 4 A2)**.

Neutral association: Bacteroides (genus: 1.02 [0.94-1.10], P=0.68) **(Figure 4 A3).**

This stratified pattern suggests taxonomic-specific modulation of host susceptibility, with Actinobacteria and Negativicutes emerging as consistent risk predictors.

### COVID-19 infection

Stage-specific microbial dynamics revealed divergent associations **(Figure 4B)**:

Positive correlates: *Class Negativicutes* (1.13 [1.02, 1.26]), *order Selenomonadales* (1.13 [1.02, 1.26]) **(Figure 4 B1)**.

Negative correlates: *genus Rum.inococcustorquesgroup* (0.54[0.39, 0.74]), *family Alcaligenaceae* (0.87 [0.78, 0.96]), *genus*. *Parasutterella* (0.90 [0.83, 0.97]), *phylun Lentisphaerae* (0.93 [0.87, 0.99]), *genus Ruminococcaceae UCG014* (0.88 [0.80, 0.97]), *genus Ruminococcus1* (0.73 [0.54, 0.99]), *genus Ruminococcaceae UCG003* (0.90 [0.82, 0.99]) **(Figure 4 B2)**.

Non-significant taxa: family Bifidobacteriaceae (1.06 [0.88, 1.29]), genus Oxalobacter (0.87 [0.74, 1.04]), family Streptococcaceae (1.12 [0.84, 1.50]), genus Eubacteriumcoprostanoligenesgroup (0.84 [0.61, 1.15]), genus Oxalobacter (0.87 [0.74, 1.04]), genus Allisonella (1.00 [0.88, 1.14]), genus Erysipelatoclostridium (0.93 [0.74, 1.16]) **(Figure 4 B3)**.

Notably, Parasutterella demonstrated dual protective roles across susceptibility (0.90 [0.84-0.97]) and infection stages (0.90 [0.83-0.97]).

### COVID-19 hospitalization

Microbial predictors of clinical deterioration **(Figure 4 C)**:

High-risk indicators: *order MollicutesRF9* (1.13 [1.04, 1.22]), *Alloprevotella* (1.25 [1.07, 1.45]), *class Actinobacteria* (1.11 [1.03, 1.18]), *family Bifidobacteriaceae* (1.54 [0.52, 4.56]), *class Negativicutes* (1.23 [1.11, 1.37]), *order Selenomonadales* (1.13 [1.03, 1.25]). At the genus level, *Dorea* (1.16 [1.05, 1.28]), *Prevotella9* (1.21 [1.04, 1.41]), and an *unidentified genus (id.1000005472)* (1.10 [1.03, 1.18]) also demonstrated positive correlations. Additionally, family-level taxa such as an *unidentified family (id.1000005471)* (1.11 [1.03, 1.21]) and *FamilyXIII* (1.30 [1.03, 1.64]), as well as *order Bacteroidales* (1.09 [1.01, 1.18]) and *genus Eubacteriumruminantiumgroup* (1.07 [1.01, 1.12]), were similarly linked to greater hospitalization severity **(Figure 4 C1)**.

Protective factors: *Ruminiclostridium6* (0.81 [0.69, 0.94]), gut production of the SCFA butyrate (0.97 [0.94-0.99]), *genus Parasutterella* (0.84 [0.72, 0.98]), *Marvinbryantia* (0.89 [0.81, 0.97]), *Tyzzerella3* (0.95 [0.91, 0.99]), *Ruminococcaceae* UC*G*014 (0.86 [0.77, 0.95]), *genus Olsenella* (0.94 [0.91, 0.97]), and* Alistipes* (0.78 [0.63, 0.96]) **(Figure 4 C2)**.

The inverse correlation between SCFA butyrate and hospitalization severity highlights potential therapeutic targets.

### COVID-19 severe

Integrated analysis of nine studies** (Figure 4D)** revealed significant gut microbiota perturbations associated with COVID-19 severity. Three distinct microbial patterns emerged through Mendelian randomization analyses.

Risk-enhancing taxonomic signatures: Phylum-level dysbiosis was characterized by increased *Actinobacteria* abundance (1.20 [1.01, 1.42]), particularly within the *class Negativicutes* (1.29 [1.08, 1.55]). Order-level alterations demonstrated consistent elevation of *Selenomonadales* (1.19 [1.01, 1.40]) and *Mollicutes RF9* (1.15 [1.07, 1.24]). Genus-level analysis identified multiple risk biomarkers, including *Alloprevotella* (1.66 [1.36, 2.01]) and *Bifidobacterium* (2.09 [1.15, 3.81]), the latter showing paradoxical associations despite its conventional probiotic role. Notably, an uncharacterized *genus (id.1000005472)* exhibited robust correlation with disease severity (1.24 [1.06, 1.44]), warranting taxonomic clarification **(Figure 4 D1)**.

Protective microbial consortia: Commensal taxa demonstrating inverse correlations with disease severity included butyrate-producing *Oxalobacter* (0.84 [0.76, 0.92]) and mucin-degrading *Subdoligranulum* (0.82 [0.76, 0.89]). The order *Lactobacillales* (0.86 [0.79, 0.95]) showed particular promise for microbial intervention, potentially through competitive exclusion mechanisms. *Cyanobacteria* at the phylum level (0.85 [0.79, 0.93]) suggested light-dependent metabolic pathways might influence disease progression **(Figure 4 D2)**.

Neutral microbial associations: Multiple taxa including *Faecalibacterium* (0.54 [0.18, 1.61]) and *Romboutsia* (1.79 [0.82, 3.87]) demonstrated non-significant associations (all P>0.05), with confidence intervals spanning protective to risk-enhancing ranges. Gut metabolites showed similar neutrality, with *butyrate* (1.01 [0.96, 1.06]) and *fecal propionate* (0.97 [0.86, 1.09]) production levels exhibiting no disease-modifying effects **(Figure 4 D3)**.

Collectively, this analysis delineates a dynamic reciprocity between gut microbiota composition and COVID-19 severity, wherein specific taxa (e.g., *Actinobacteria*, *Negativicutes*) demonstrate disease-aggravating effects, while others (*Oxalobacter*, *Parasutterella*) confer protection through immunomodulatory pathways, alongside commensal taxa exhibiting neutral disease associations.

### Publication bias

**Figure 5:**
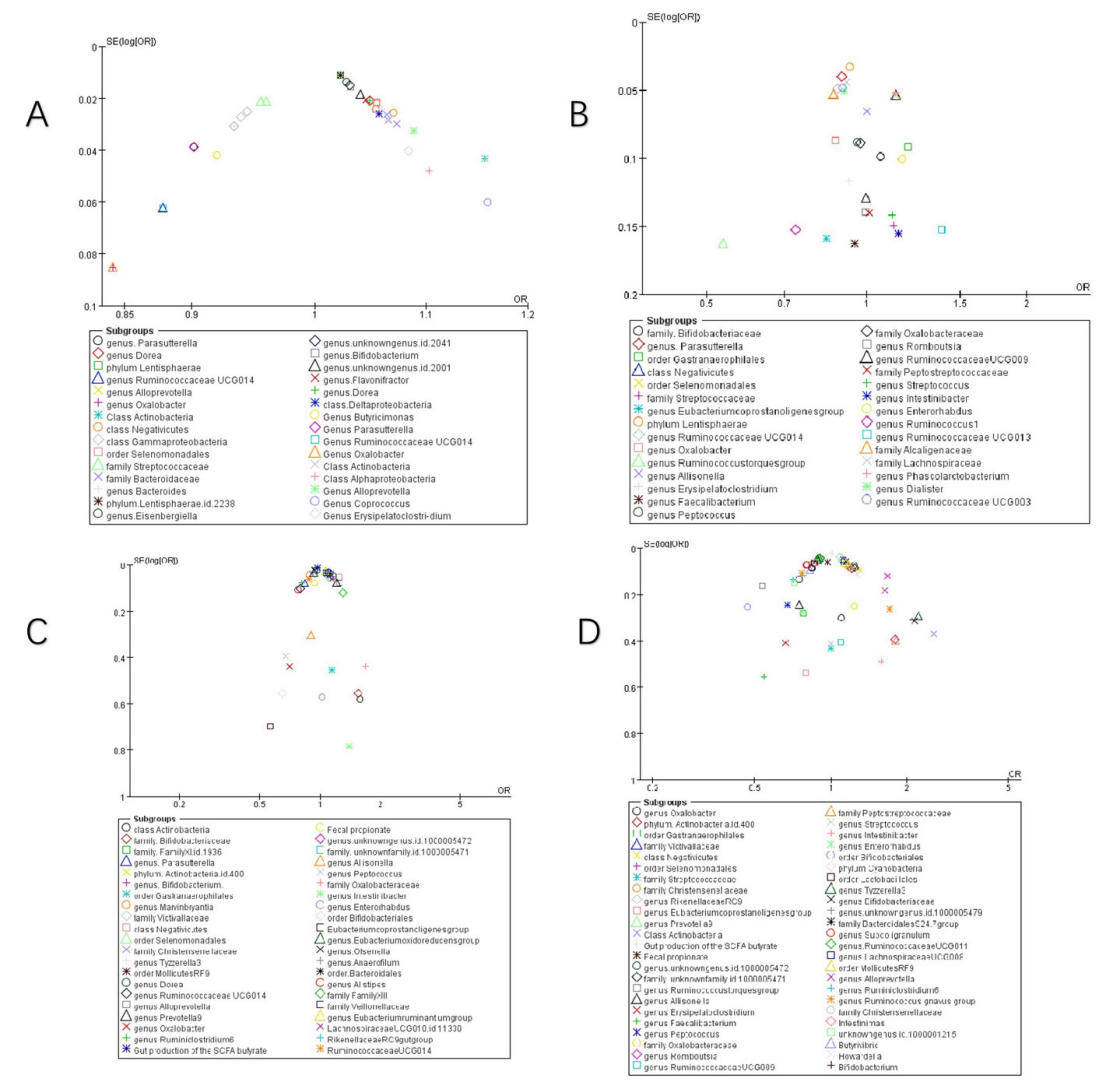
**Funnel plot of summary of gut microbiota of different COVID-19 severities. A)** COVID-19 susceptibility; **B)** COVID-19 infection; **C)** COVID-19 hospitalization; **D)** COVID-19 severe.

Funnel plots assessed publication bias in pneumonia studies. COVID-19 analyses demonstrated acceptable bias ranges for suspected cases **(Figure 5 A)**, hospitalizations **(Figure 5 C)**, and severe outcomes **(Figure 5 D)**, evidenced by symmetrical distribution of studies near the funnel apex. Conversely, COVID-19 infection studies **(Figure 5 B)** exhibited significant publication bias, potentially due to limited included reports. Egger's regression modeled the logarithm of effect size against standard error, accounting for sample size influences on estimation precision. Results indicated: no significant publication bias for hospitalizations (t = 1.296, df = 42, p = 0.202; β = -0.019, 95% CI: -0.061 to 0.023), severe cases (t = 0.517, df = 65, p = 0.607; β = -0.037, 95% CI: -0.105 to 0.029), infections (t = 0.585, df = 22, p = 0.565; β = -0.089, 95% CI: -0.180 to 0.003), or susceptibility (t = -0.859, df = 39, p = 0.396; β = 0.039, 95% CI: 0.003 to 0.075). Minimal funnel asymmetry and nonsignificant intercept terms support robust pooled estimates.

## Discussion

The current body of research, encompassing data from approximately 32, 748, 274 participants, has demonstrated a significant association between COVID-19 (new coronary pneumonia) and intestinal microbiota. An increasing number of scientific studies have progressively elucidated the role of gut microbiota in the development and progression of COVID-19 28-30. This study is the first to employ Mendelian randomization (MR) to systematically summarize these findings. The MR approach assumes that the observed associations are independent of traditional confounding factors, thereby providing a robust framework for causal inference.

Our analysis incorporated data from 11 studies, involving over 32, 748, 274 participants and 1, 487 single nucleotide polymorphisms (SNPs), to investigate the causal relationship between intestinal microbiota, its metabolites, and COVID-19. Among these studies, two focused on COVID-19 infection, four on COVID-19 susceptibility, nine on COVID-19 severe, six on COVID-19 hospitalization, and others on related conditions, including bacterial pneumonia (BP), pneumococcal pneumonia (PP), and bronchopneumonia or lung abscess **(Table S3)**. The analysis revealed that certain microbial taxa, such as *order Bifidobacteriales, genus Ruminococcustorquesgroup, genus Ruminiclostridium6, genus Oxalobacter, genus Ruminococcaceae UCG014*, *genus Olsenella*, *genus Tyzzerella3*, *genus Subdoligranulum*, *family Christensenellaceae*, *phylum Cyanobacteria*, *order Lactobacillales*, class *Gammaproteobacteria,* genus *Anaerofilum*, *genus Parasutterella*, and *family Streptococcaceae*, were negatively correlated with an increased risk of pneumonia. Conversely, other taxa, such as *class Actinobacteria*, *genus Prevotella 9*, *genus Alloprevotella*, *genus Lachnospiraceae UCG008*, *genus Rikenellaceae RC9*, *order M*ollicutes *RF9*, *genus Bacteroides*, and *family Bacteroidaceae*, were positively correlated with an increased risk of pneumonia. Interestingly, the *order Gastroaerophilales* exhibited an inconspicuous association with pneumonia. These findings suggest that the causal relationship between gut microbiota and pneumonia, including COVID-19, may become clearer as more MR studies are conducted.

The gut microbiota critically regulates spatiotemporal dynamics of mucosal immune homeostasis, demonstrating pathogen-specific immunomodulatory patterns in bacterial, viral, and fungal pneumonias 31. This microbial regulation directly influences host immune responses according to pathogen class. Notably, COVID-19-induced gut dysbiosis correlates with neuropsychiatric sequelae 32-35 and exacerbates disease severity in obese NASH patients, where *Peptococcus* abundance associates with pro-inflammatory signatures in pulmonary and hepatic tissues 36. Although *Streptococcus* enrichment occurs in COVID-19 cohorts 37 - contrasting with our findings **(Table S3)** - emerging evidence implicates elevated streptococcal loads in upregulating viral entry receptors, potentially facilitating infection 38. Similarly, anaerobic genera such as *Prevotella* and *Veillonella* may propagate under hypoxia, potentially contributing to secondary pulmonary infections 39.

Consistent with our data, multiple studies report reduced α-diversity in COVID-19 fecal microbiomes 40-43, diminished SCFA-producing taxa (e.g., *Ruminococcaceae*), and decreased *Ruminococcus* abundance 42, 44. Gut-derived SCFAs, particularly butyrate, translocate hematogenously to the pulmonary compartment where they enhance alveolar macrophage bactericidal capacity while suppressing pro-inflammatory cytokine cascades 31. Clinically, severe COVID-19 exhibits marked depletion of butyrogenic bacteria including *Faecalibacterium* 34. Therapeutic *Bifidobacterium* supplementation partially restores microbial diversity, fortifies intestinal barrier integrity, and demonstrates efficacy against mycoplasma pneumonia 35. A paradoxical positive association (odds ratio [OR] > 2.0) was observed for the Bifidobacterium genus, contradicting prior expectations 35. This finding may be attributable to genetic pleiotropy, wherein genetic variants underlying the instrumental variables could directly influence COVID-19 severity through alternative biological mechanisms, independent of their effects on microbial abundance. Furthermore, microbial impacts are likely highly context-dependent, modulated by specific host immune status and environmental context.

Furthermore, COVID-19 severity inversely correlates with *Actinomycetota* abundance but positively associates with *Bacteroidetes* prevalence, alongside pandemic-associated shifts in antimicrobial resistance genes 37. Critically, *Ruminococcus* torques group abundance confers reduced infection risk, while *Bifidobacteriales* enrichment predicts lower severe disease incidence 44 - observations aligning with our dataset. Collectively, these findings position the gut microbiota as a viable target for COVID-19 adjuvant therapies 45.

Given the extensive assessment of gut microbial taxa in this study, potential false-positive associations may arise due to multiple testing. Furthermore, as the primary GWAS summary statistics were derived from European ancestry cohorts, the generalizability of findings to other populations is constrained. Future replication studies in more diverse ethnic populations are required to validate these associations.

While Mendelian randomization analyses cannot establish definitive causality and remain limited by cohort sizes, they provide compelling evidence for gut microbiota-COVID-19 interactions. This synthesis resolves taxonomic controversies and advances evidence-based medicine, suggesting that clinical microbiota modulation may improve COVID-19 prognoses. Further mechanistic investigations are warranted to translate these associations into therapeutic strategies.

## Conclusion

In summary, we conducted a comprehensive assessment to estimate the causal relationship between gut microbiota and COVID-19. Our meta-analysis incorporated data from 11 studies, encompassing over 32, 748, 274 participants and 1, 487 single nucleotide polymorphisms (SNPs). This analysis identified potential causal links between 52 types of gut microbiota, two gut microbiota metabolites, and four levels of COVID-19 severity. These findings contribute to a deeper understanding of the role of gut microbiota in the progression of COVID-19 and aim to provide an evidence-based foundation for exploring the interplay between the gut microbiome and the disease.

## Supplementary Material

Supplementary tables.

## Figures and Tables

**Table 1:** Methods to assess the quality of MR studies

Item	Grade	Criteria
A. Full IV analysis	Good	Full IV analyses, such as two-stage least-squares regression for one-sample MR study andinverse-variance weighted method for two-sample MR study.
	Poor	Without full IV analyses; only uses other approaches, such as an association analysis betweerthe genetic variant and outcome.
B.Relevance assumption	Good	For one-sample R study, the assumption is tested by reporting an F-statistic (F>10); for twosample MR study, strongly and robustly associated SNPs from GWAS are selected (P<5x10-8).
	Moderate	Associated SNPs are selected using a P value threshold not satisfying Bonferroni correction
	Poor	Failure to describe whether the assumption is satisfied.
C. Independence assumption and exclusion restriction assumption	Good	Assumption is tested using MR-Egger regression, MR-Pleiotropy Residual Sum and Outlier, and other noyel methods
	Moderate	Full IV analysis is selected based on literature research and without testing the assumption.
	Poor	Failure to describe whether the assumption is satisfied.
D. Sensitivity analysis	Good	Sensitivity analysis is conducted, and results that are consistent with primary analyses are reported.
	Poor	Failed sensitivity analysis or inconsistent results are reported.
E.Population stratification	Good	Absence of population stratification.
	Poor	Presence of population stratification or failure to report population information.
F. Non-linearity correlation	Good	Potential non-linearity correlations of exposure and outcome variables are explored.
	Poor	Failure to describe potential non-linearity correlations.

**Table 2:** Baseline characteristic of the included literature

Label	Disease	Case	Sample	Bacterial flora/Metabolites	SNP Quantities	OR (95%CI) IVW Results	Remark
Yingjian Liu 2024 17	Pneumonia	/	456348	genus.Anaerofilum.id.2053	7	1.34(1.04,1.72)	IVW
				family.Bifidobacteriaceae.id.433	13	0.68(0.54,0.85)	IVW
				family.Coriobacteriaceae.id.811	17	1.34(1,1.81)	IVW
				order.Coriobacteriales.id.810	17	1.34(1,1.81)	IVW
				class.Coriobacteriia.id.809	17	1.34(1,1.81)	IVW
				family.FamilyXI.id.1936	8	1.18(1.01,1.38)	IVW
				genus.LachnospiraceaeND3007group.id.11317	3	2.1(1.17,3.78)	IVW
	BP(bacterial pneumonia)	/	456348	genus.Parasutterella.id.2892	12	2.75(1.49,5.08)	IVW
				phylum.Actinobacteria.id.400	16	2.09(1.13,3.88)	IVW
				family.Bifidobacteriaceae.id.433	15	2.05(1.17,3.58)	IVW
				genus.Bifidobacterium.id.436	16	1.93(1.15,3.22)	IVW
				family.Enterobacteriaceae.id.3469	7	3.39(1.35,8.48)	IVW
				order.Enterobacteriales.id.3468	7	3.39(1.35,8.48)	IVW
				order.Gastranaerophilales.id.1591	9	1.67(1.02,2.74)	IVW
				family.Rhodospirillaceae.id.2717	14	0.55(0.33,0.91)	IVW
				order.Rhodospirillales.id.2667	13	0.56(0.33,0.95)	IVW
	BLA(bronchopneumonia、lung abscess)	/	456348	genus.Odoribacter.id.952	3	0.17(0.03,0.97)	IVW
				genus.Paraprevotella.id.962	12	0.54(0.3,0.94)	IVW
				phylum.Bacteroidetes.id.905	11	0.32(0.11,0.92)	IVW
				genus.ChristensenellaceaeR.7group.id.11283	9	0.25(0.09,0.73)	IVW
				genus.Fusicatenibacter.id.11305	18	2.2(1.03,4.7)	IVW
				genus.Marvinbryantia.id.2005	9	0.41(0.17,1)	IVW
				class.Methanobacteria.id.119	10	1.58(1,2.5)	IVW
				family.Methanobacteriaceae.id.121	10	1.58(1,2.5)	IVW
				order.Methanobacteriales.id.120	10	1.58(1,2.5)	IVW
				genus.Methanobrevibacter.id.123	6	1.91(1.05,3.47)	IVW
				family.Peptococcaceae.id.2024	10	2.03(1.01,4.07)	IVW
				family.Porphyromonadaceae.id.943	9	4.93(1.2,20.15)	IVW
	PP(pneumococcal pneumonia)	/	456348	genus.Adlercreutzia.id.812	5	0.74(0.56,0.97)	IVW
				genus.Holdemanella.id.11393	10	1.2(1.02,1.41)	IVW
				genus.Lachnospira.id.2004	6	0.66(0.47,0.93)	IVW
				genus.LachnospiraceaeNC2004group.id.11316	9	0.77(0.65,0.91)	IVW
				family.Rikenellaceae.id.967	21	1.31(1.1,1.57)	IVW
Tian, Siyu 2024 18	COVID-19 infection	112612	2474079				
	COVID-19 hospitalization	24274	2061529	Bifidobacterium.id.436	13	1.126(1.021-1.242)	IVW
				LachnospiraceaeUCG010.id.11330	10	1.139(1.009-1.287)	IVW
				RikenellaceaeRC9gutgroup.id.11191	13	1.081(1.019-1.147)	IVW
				RuminococcaceaeUCG014.id.11371	11	0.822(0.782-0.995)	IVW
	COVID-19 severity	8779	1001875	Intestinimas.id.2062	16	1.179(1.006-1.383)	IVW
Yuxin Zou 2024 19	COVID-19 severity	13769	1072442	Victivallaceae,	12	0.888 (0.801-0.984)	IVW
Weifeng Shang 2023 20	COVID-19 susceptibility	159 840.00	2782977	class Negativicutes	12	1.05(1.01-1.10)	IVW
				class Gammaproteobacteria	7	0.94( 0.89-0.99)	IVW
				order Selenomonadales	12	1.05(1.01-1.10)	IVW
				family Streptococcaceae	14	0.95(0.92-1.00)	IVW
				family Bacteroidaceae	9	1.06( 1.01-1.12)	IVW
				genus Bacteroides	9	1.06(1.01-1.12)	IVW
	COVID-19 severity	18 152.00	1145546	phylum Cyanobacteria	8	0.85(0.76-0.96)	IVW
				order Lactobacillales	15	0.87(0.76-0.98)	IVW
				family Christensenellaceae	11	0.87(0.77-0.99)	IVW
				genus Subdoligranulum	11	0.8(0.69-0.92)	IVW
				genus Tyzzerella3	13	0.89(0.81-0.97)	IVW
				genus RuminococcaceaeUCG011	8	0.91(0.83-0.99)	IVW
				order MollicutesRF9	13	1.14(1.01-1.29)	IVW
				genus RikenellaceaeRC9	11	1.09(1.01-1.17)	IVW
				genus LachnospiraceaeUCG008	11	1.12(1.00-1.26)	IVW
Meng-Mei Zhong 2023 21	COVID-19 susceptibility	159840	2782977	class Gammaproteobacteria	6	0.933(0.879-0.991)	IVW
				family Streptococcaceae	14	0.955(0.916-0.995)	IVW
				class Negativicutes	13	1.054(1.005-1.105)	IVW
				order Selenomonadales	12	1.054(1.005-1.105)	IVW
				family Bacteroidaceae	9	1.06(1.007-1.125)	IVW
				genus Bacteroides	9	1.064(1.007-1.125)	IVW
	COVID-19 severity	18152	1145546	phylum Cyanobacteria	8	0.852(0.760-0.955)	IVW
				order Lactobacillales	15	0.867(0.764-0.983)	IVW
				genus RuminococcaceaeUCG011	11	0.907(0.832-0.988)	IVW
				genus Subdoligranulum	11	0.807(0.699-0.932)	IVW
				genus Tyzzerella3	13	0.885(0.810-0.967)	IVW
				order MollicutesRF9	13	1.141(1.009-1.291)	IVW
				genus RikenellaceaeRC9	8	1.085(1.009-1.167)	IVW
	COVID-19 hospitalization	44986	2356386	genus Marvinbryantia	10	0.886(0.812-0.967)	IVW
				genus Olsenella	11	0.942(0.897-0.990)	IVW
				family Veillonellaceae	19	1.069(1.002-1.140)	IVW
				genus Eubacteriumruminantiumgroup	18	1.065(1.010-1.123)	IVW
				genus Dorea	10	1.162(1.055-1.279)	IVW
Zengbin Li 2023 22	COVID-19 infection	38984	1644784	phylum Lentisphaerae	9	0.93(0.87-0.99)	IVW
				family Alcaligenaceae	12	0.87(0.78-0.96)	IVW
				family Lachnospiraceae	17	0.91(0.84-1.00)	IVW
				genus Dialister	11	0.91(0.82-1.00)	IVW
				genus Parasutterella	14	0.89(0.83-0.97)	IVW
				genus Ruminococcaceae UCG003	12	0.90(0.82-0.99)	IVW
				genus Ruminococcaceae UCG014	11	0.88(0.80-0.97)	IVW
				class Negativicutes	12	1.13(1.02-1.26)	IVW
				order Selenomonadales	12	1.13(1.02-1.26)	IVW
				genus Phascolarctobacterium	9	1.13(1.02-1.25)	IVW
	COVID-19 hospitalization	9986	1877672	genus Alistipes	14	0.78(0.63-0.96)	IVW
				genus Parasutterella	14	0.84(0.72-0.98)	IVW
				genus Ruminiclostridium6	15	0.80(0.69-0.94)	IVW
				genus Ruminococcaceae UCG014	11	0.79(0.65-0.97)	IVW
				family Family XIII	10	1.30(1.03-1.64)	IVW
				family Victivallaceae	12	1.11(1.00-1.24)	IVW
				genus Alloprevotella	5	1.25(1.07-1.45)	IVW
				genus Prevotella9	14	1.21(1.04-1.41)	IVW
	COVID-19 severity	5101	1383241	genus Ruminococcus gnavus group	12	0.77(0.62-0.95)	IVW
				genus Oxalobacter	11	0.84(0.71-1.00)	IVW
				genus Ruminiclostridium6	16	0.78(0.62-0.98)	IVW
				genus Alloprevotella	5	1.67(1.32-2.11)	IVW
Han Chen 2023 23	COVID-19 susceptibility	38984	1644784	Genus Butyricimonas	13	0.919(0.847-0.998)	IVW
				Genus Parasutterella	16	0.902(0.836-0.973)	IVW
				Genus Ruminococcaceae UCG014	9	0.878(0.777-0.992)	IVW
				Genus Oxalobacter	13	0.842(0.712-0.994)	IVW
				Class Actinobacteria	21	1.156(1.062-1.258)	IVW
				Class Alphaproteobacteria	9	1.102(1.004-1.211)	IVW
				Genus Alloprevotella	7	1.088(1.021-1.160)	IVW
				Genus Coprococcus	10	1.159(1.030-1.304)	IVW
				Genus Erysipelatoclostri-dium	13	1.083(1.001-1.172)	IVW
	COVID-19 severity	5101	1383241	Genus Oxalobacter	13	0.842 (0.712-0.994)	IVW
Wanqiang Lv 2023 24	COVID-19 hospitalization	6406	902088	Gut production of the SCFA butyrate	8	0.96832539912073(0.94416699163366-0.993101947950892)	IVW
				Fecal propionate	3	0.941469863248343(0.808083479895778-1.09687368379214)	IVW
	COVID-19 severity	3886	622265	Gut production of the SCFA butyrate	7	1.00845602976638(0.96356320994773-1.05544042515628)	IVW
				Fecal propionate	3	0.968794000309792(0.857951748844421-1.09395640990347)	IVW
Jukun Song 2023 25	COVID-19 susceptibility	/	159840	class.Gammaproteobacteria.id.3303	10	0.943826(0.898701-0.991217)	IVW
				phylum.Lentisphaerae.id.2238	15	1.021896(1.00006-1.044209)	IVW
				genus.Eisenbergiella.id.11304	12	1.027563(1.000392-1.055472)	IVW
				genus.unknowngenus.id.2041	13	1.03016(1.000408-1.060798)	IVW
				genus.Bifidobacterium.id.436	21	1.030965(1.000158-1.062721)	IVW
				genus.unknowngenus.id.2001	11	1.039753(1.002465-1.078429)	IVW
				genus.Flavonifractor.id.2059	10	1.044181(1.002618-1.087467)	IVW
				genus.Dorea.id.1997	14	1.048128(1.006111-1.091899)	IVW
				order.Selenomonadales.id.2165	15	1.053979(1.010557-1.099266)	IVW
				class.Deltaproteobacteria.id.3087	13	1.055903(1.003375-1.11118)	IVW
				genus.Bacteroides.id.918	12	1.059099(1.010079-1.110498)	IVW
				class.Negativicutes.id.2164	11	1.069307(1.016779-1.124549)	IVW
				family.Bacteroidaceae.id.917	8	1.072539(1.011616-1.137131)	IVW
	COVID-19 hospitalization	/	44986	family.Christensenellaceae.id.1866	13	0.918613(0.846107-0.997332)	IVW
				genus.Eubacteriumoxidoreducensgroup.id.11339	9	0.934016(0.87255-0.999811)	IVW
				genus.Olsenella.id.822	13	0.938009(0.896461-0.981482)	IVW
				genus.Anaerofilum.id.2053	12	0.945835(0.896604-0.997769)	IVW
				genus.Tyzzerella3.id.113.35	18	0.951996(0.910977-0.994862)	IVW
				family.FamilyXI.id.1936	12	0.95861(0.919248-0.999658)	IVW
				order.Bacteroidales.id.913	16	1.092775(1.01484-1.176694)	IVW
				genus.unknowngenus.id.1000005472	15	1.101318(1.028715-1.179046)	IVW
				class.Actinobacteria.id.419	21	1.104484(1.031225-1.182947)	IVW
				family.unknownfamily.id.1000005471	12	1.11355(1.026157-1.208386)	IVW
				phylum.Actinobacteria.id 400	20	1.121487(1.028754-1.22258)	IVW
				order.MollicutesRF9.id.11579	12	1.126898(1.044468-1.215834)	IVW
				order.Selenomonadales.id.2165	15	1.134561(1.026603-1.253872)	IVW
				class.Negativicutes.id.2164	11	1.234846(1.112774-1.37031)	IVW
	COVID-19 severity	/	18152	genus.Subdoligranulum.id.2070	13	0.855125(0.749524-0.975604)	IVW
				genus.Tyzzerella3.id.11335	14	0.896826(0.82266-0.97768)	IVW
				genus.RuminococcaceaeUCG011.id.11368	8	0.906709(0.832425-0.987621)	IVW
				genus.Prevotella9.id.11183	19	1.108017(1.015604-1.208839)	IVW
				genus.LachnospiraceaeUCG008.id.11328	12	1.110538(1.000231-1.233009)	IVW
				family.BacteroidalesS24.7group.id.11173	10	1.149522(1.02712-1.28651)	IVW
				genus.unknowngenus.id.1000005479	6	1.173132(1.004522-1.370044)	IVW
				order.Selenomonadales.id.2165	12	1.188812(1.01203-1.396475)	IVW
				phylum.Actinobacteria.id.400	17	1.202516(1.015075-1.42457)	IVW
				family.unknownfamily.id.1000005471	11	1.23367(1.047721-1.452621)	IVW
				genus.unknowngenus.id.1000005472	11	1.237166(1.064543-1.437781)	IVW
				class.Negativicutes.id.2164	8	1.292966(1.075591-1.554273)	IVW
				order.MollicutesRF9.id 11579	15	1.168451(1.017332-1.342018)	IVW
Hanyu Zhang 2023 26	COVID-19 infection	38984	1644784	genus Ruminococcustorquesgroup	1	0.537(0.391-0.738)	IVW
				genus Ruminococcaceae UCG013	1	1.38206616435633(1.025-1.863)	IVW
				genus Ruminococcus1	1	0.734645873967539(0.545-0.99)	IVW
				*genus Allisonella*	1	0.999477974302852(0.879-1.137)	IVW
				genus Eubacteriumcoprostanoligenesgroup	1	0.839701170368478(0.615-1.146)	IVW
				genus Oxalobacter	1	0.872561011613187(0.736-1.035)	IVW
				genus Erysipelatoclostridium	1	0.925967316791888(0.737-1.163)	IVW
				genus Faecalibacterium	1	0.947829609211915(0.69-1.303)	IVW
				genus Peptococcus	1	0.957340664124295(0.805-1.138)	IVW
				family Oxalobacteraceae	1	0.973361241524337(0.818-1.159)	IVW
				genus Romboutsia	1	0.991370771376931(0.755-1.302)	IVW
				genus RuminococcaceaeUCG009	1	0.997685345952551(0.774-1.285)	IVW
				family Peptostreptococcaceae	1	1.00904062177387(0.767-1.328)	IVW
				genus Bifidobacteriaceae	1	1.06235820628227(0.875-1.29)	IVW
				genus Streptococcus	1	1.11442825766029(0.844-1.471)	IVW
				family Streptococcaceae	1	1.12075212488415(0.837-1.501)	IVW
				genus Intestinibacter	1	1.14628731724782(0.846-1.553)	IVW
				*genus Enterorhabdus*	1	1.1672334778462(0.958-1.422)	IVW
				order Gastranaerophilales	1	1.19602074416788(0.999-1.432)	IVW
	COVID-19 hospitalization	3159	7206	Eubacteriumcoprostanoligenesgroup	1	0.568684261257267(0.145-2.223)	IVW
				Bifidobacteriales	1	0.648560491804976(0.219-1.917)	IVW
				genus Peptococcus	1	0.675976747986784(0.313-1.459)	IVW
				Oxalobacter	1	0.706206493883378(0.3-1.661)	IVW
				*Allisonella*	1	0.903849603717244(0.499-1.636)	IVW
				*Enterorhabdus*	1	1.02698051702283(0.335-3.146)	IVW
				Gastranaerophilales	1	1.14110831926724(0.47-2.773)	IVW
				Intestinibacter	1	1.38991028236733(0.301-6.42)	IVW
				Bifidobacteriaceae	1	1.54120056443399(0.521-4.56)	IVW
				class Actinobacteria	1	1.57493389505008(0.504-4.92)	IVW
				family Oxalobacteraceae	1	1.68539507129741(0.716-3.969)	IVW
	COVID-19 severity	5101	1383241	*order Bifidobacteriales*	2	0.471(0.286-0.774)	IVW
				genus Ruminococcustorquesgroup	1	0.536877354869706(0.391-0.738)	IVW
				genus Bifidobacteriaceae	2	2.124(1.152-3.915)	IVW
				*genus Tyzzerella3*	1	2.21142565432121(1.246-3.924)	IVW
				*class Actinobacteria*	1	2.53280022758574(1.228-5.224)	IVW
				*genus Faecalibacterium*	1	0.545239789689792(0.184-1.614)	IVW
				*genus Erysipelatoclostridium*	1	0.66116800731294(0.298-1.468)	IVW
				*genus Peptococcus*	1	0.675101722137951(0.419-1.088)	IVW
				*genus Allisonella*	1	0.750932133107426(0.464-1.215)	IVW
				*genus Enterorhabdus*	1	0.76744596953411(0.447-1.317)	IVW
				order Gastranaerophilales	1	0.7795799733847(0.45-1.35)	IVW
				*genus Eubacteriumcoprostanoligenesgroup*	1	0.792819896331787(0.277-2.271)	IVW
				*family Streptococcaceae*	1	0.999972600375377(0.429-2.332)	IVW
				*genus Streptococcus*	1	0.999973966838869(0.447-2.235)	IVW
				*genus RuminococcaceaeUCG009*	1	1.09160755405964(0.494-2.414)	IVW
				*genus Oxalobacter*	1	1.09557314891857(0.606-1.981)	IVW
				*family Oxalobacteraceae*	1	1.23244499853025(0.755-2.012)	IVW
				*genus Intestinibacter*	1	1.57226695768584(0.604-4.094)	IVW
				*genus Romboutsia*	1	1.78794246889422(0.825-3.874)	IVW
				*family Peptostreptococcaceae*	1	1.79858455998767(0.824-3.924)	IVW
Han Yan 2023 27	COVID-19 severity	5101	1383241	Ruminiclostridium6	14	0.708(0.544-0.921)	IVW(if the IV number was more than 1)
				unknowngenus.id.1000001215	5	0.72(0.536-0.966)	IVW(if the IV number was more than 1)
				Oxalobacter	4	0.752(0.578-0.98)	IVW(if the IV number was more than 1)
				Butyrivibric	14	0.83(0.69-1.000)	IVW(if the IV number was more than 1)
				Oxalobacter	11	0.842(0.709-1.000)	IVW(if the IV number was more than 1)
				Howardella	7	1.264(1.009-1.583)	IVW(if the IV number was more than 1)
				Alloprevotella	4	1.627(1.14-2.323)	IVW(if the IV number was more than 1)
				Ruminococcus gnavus group	2	1.703(1.018-2.849)	IVW(if the IV number was more than 1)
				Bifidobacterium	2	2.092(1.149-3.808)	IVW(if the IV number was more than 1)

**Table 3:** Consolidated Effects of Key Taxa on Pneumonia Severity

Taxon	Studies	WMD (95% CI)	P-value	Heterogeneity (I²)	Direction
Phylum Actinobacteria.id	3	1.14 (1.05--1.23)	0.001	53%	↑ Severity
Class Negativicutes	6	1.07 (1.04--1.11)	<0.0001	34%	↑ Severity
Class Actinobacteria	4	1.15 (1.08--1.22)	<0.00001	42%	↑ Severity
Order MollicutesRF9	4	1.14 (1.08--1.20)	<0.00001	0%	↑ Severity
Order Selenomonadales	6	1.07 (1.04--1.09)	<0.00001	6%	↑ Severity
Family Streptococcaceae	4	0.96 (0.93--0.99)	0.005	0%	↓ Severity
Family Bacteroidaceae	3	1.06 (1.03--1.09)	<0.0001	0%	↑ Severity
Genus Tyzzerella3	5	0.92 (0.88--0.96)	<0.0001	24%	↓ Severity
Genus Oxalobacter	8	0.84 (0.78--0.91)	<0.001	0%	↓ Severity
Genus Parasutterella	4	0.89 (0.85--0.94)	<0.0001	0%	↓ Severity
Genus RuminococcaceaeUCG014	3	0.87 (0.81--0.93)	<0.0001	0%	↓ Severity
Genus RuminococcaceaeUCG011	3	0.91 (0.86--0.95)	0.0002	0%	↓ Severity
Genus Subdoligranulum	3	0.80 (0.76--0.85)	<0.00001	0%	↓ Severity
Genus Alloprevotella	4	0.89 (0.85--0.94)	0.005	83%	↑ Severity
Genus RikenellaceaeRC9	3	1.08 (1.04--1.13)	0.0001	0%	↑ Severity
Genus Bacteroides	3	1.06 (1.03--1.09)	<0.0001	0%	↑ Severity
Genus Bifidobacterium	4	0.89 (0.85--0.94)	0.001	99%	↑ Severity
