# Supplementary material for: Association Between Gut Microbiota and Pneumonia Risk: A Systematic Review and Mendelian Randomization
Source: Int J Med Sci. 2025 Jul 28;22(14):3511–27. doi: 10.7150/ijms.114372 (PMC12434694; doi:10.7150/ijms.114372)
Supplement: Supplementary file 1 — Supplementary tables. [file ijmsv22p3511s1.pdf]

Supplementary Table S1 Search strategies and results in each database. Detailed search strategies and results across multiple databases (Embase, PubMed, Cochrane Library, Web of Science, and Scopus) for studies investigating the relationship between gut microbiota and COVID-19 outcomes.

Supplementary Table S2 Summary table of the impact of different bacterial taxa and metabolites on COVID-19 susceptibility, infection, hospitalization, and severity, including odds ratios (OR) with 95% confidence intervals (CI) from Mendelian randomization (MR) analyses.

Supplementary Table S3 Summary of bacterial flora of COVID-19 with different severities. Comprehensive summary of bacterial flora associated with COVID-19 outcomes stratified by disease severity, including MR results for specific microbial genera, families, and orders.

Supplementary Table S1 Search strategies and results in each database

Database	Search number	Search terms	Search results
**(a)Embase**	1	coronavirus disease 2019'/exp	401,460
	2	'intestine flora'/exp	110,919
	3	'mendelian randomization analysis'/exp	11,844
	4	covid 19':ti,ab,kw OR '2019-ncov infection':ti,ab,kw OR '2019 ncov infection':ti,ab,kw OR '2019-ncov infections':ti,ab,kw OR 'infection, 2019-ncov':ti,ab,kw OR 'sars-cov-2 infection':ti,ab,kw OR 'infection, sars-cov-2':ti,ab,kw OR 'sars cov 2 infection':ti,ab,kw OR 'sars-cov-2 infections':ti,ab,kw OR '2019 novel coronavirus disease':ti,ab,kw OR '2019 novel coronavirus infection':ti,ab,kw OR 'covid-19 virus infection':ti,ab,kw OR 'covid 19 virus infection':ti,ab,kw OR 'covid-19 virus infections':ti,ab,kw OR 'infection, covid-19 virus':ti,ab,kw OR 'virus infection, covid-19':ti,ab,kw OR 'covid19':ti,ab,kw OR 'coronavirus disease 2019':ti,ab,kw OR 'disease 2019, coronavirus':ti,ab,kw OR 'coronavirus disease-19':ti,ab,kw OR 'coronavirus disease 19':ti,ab,kw OR 'severe acute respiratory syndrome coronavirus 2 infection':ti,ab,kw OR 'covid-19 virus disease':ti,ab,kw OR 'covid 19	433,886

	virus disease':ti,ab,kw OR 'covid-19 virus diseases':ti,ab,kw OR 'disease, covid-19 virus':ti,ab,kw OR 'virus disease, covid-19':ti,ab,kw OR 'sars coronavirus 2 infection':ti,ab,kw OR '2019-ncov disease':ti,ab,kw OR '2019 ncov disease':ti,ab,kw OR '2019-ncov diseases':ti,ab,kw OR 'disease, 2019-ncov':ti,ab,kw OR 'covid-19 pandemic':ti,ab,kw OR 'covid 19 pandemic':ti,ab,kw OR 'pandemic, covid-19':ti,ab,kw OR 'covid-19 pandemics':ti,ab,kw

5	gastrointestinal microbiomes':ti,ab,kw OR 'microbiome, gastrointestinal':ti,ab,kw OR 'gut microbiome':ti,ab,kw OR 'gut microbiomes':ti,ab,kw OR 'microbiome, gut':ti,ab,kw OR 'gut microflora':ti,ab,kw OR 'microflora, gut':ti,ab,kw OR 'gut microbiota':ti,ab,kw OR 'gut microbiotas':ti,ab,kw OR 'microbiota, gut':ti,ab,kw OR 'gastrointestinal flora':ti,ab,kw OR 'flora, gastrointestinal':ti,ab,kw OR 'gut flora':ti,ab,kw OR 'flora, gut':ti,ab,kw OR 'gastrointestinal microbiota':ti,ab,kw OR 'gastrointestinal microbiotas':ti,ab,kw OR 'microbiota, gastrointestinal':ti,ab,kw OR 'gastrointestinal microbial community':ti,ab,kw OR 'gastrointestinal microbial communities':ti,ab,kw OR 'microbial community, gastrointestinal':ti,ab,kw OR 'gastrointestinal microflora':ti,ab,kw OR 'microflora, gastrointestinal':ti,ab,kw OR 'gastric microbiome':ti,ab,kw OR 'gastric microbiomes':ti,ab,kw OR 'microbiome, gastric':ti,ab,kw OR 'intestinal microbiome':ti,ab,kw OR 'intestinal microbiomes':ti,ab,kw OR 'microbiome, intestinal':ti,ab,kw OR 'intestinal	106,240

		microbiota':ti,ab,kw OR 'intestinal microbiotas':ti,ab,kw OR 'microbiota, intestinal':ti,ab,kw OR 'intestinal microflora':ti,ab,kw OR 'microflora, intestinal':ti,ab,kw OR 'intestinal flora':ti,ab,kw OR 'flora, intestinal':ti,ab,kw OR 'enteric bacteria':ti,ab,kw OR 'bacteria, enteric':ti,ab,kw	
	6	'mendelian randomization':ti,ab,kw	11,059
	7	#3 OR #6	13,193
	8	#2 OR #5	141,900
	9	#1 OR #4	487,122
	10	#7 AND #8 AND #9	17
**(b)PubMed**	1	"COVID-19"[MeSH Terms]	263,433
	2	"Gastrointestinal Microbiome"[MeSH Terms]	43,684
	3	"Mendelian Randomization Analysis"[MeSH Terms]	5,829
	4	"COVID-19"[Title/Abstract] OR "2019 ncov infection"[Title/Abstract] OR "2019 ncov infection"[Title/Abstract] OR "2019 ncov infections"[Title/Abstract] OR "infection 2019 ncov"[Title/Abstract] OR "sars cov 2 infection"[Title/Abstract] OR "infection	383,985

		sars cov 2"[Title/Abstract] OR "sars cov 2 infection"[Title/Abstract] OR "sars cov 2 infections"[Title/Abstract] OR "2019 novel coronavirus disease"[Title/Abstract] OR "2019 novel coronavirus infection"[Title/Abstract] OR "covid 19 virus infection"[Title/Abstract] OR "covid 19 virus infection"[Title/Abstract] OR "covid 19 virus infections"[Title/Abstract] OR "infection covid 19 virus"[Title/Abstract] OR "virus infection covid 19"[Title/Abstract] OR "coronavirus disease 2019"[Title/Abstract] OR "disease 2019 coronavirus"[Title/Abstract] OR "coronavirus disease 19"[Title/Abstract] OR "coronavirus disease 19"[Title/Abstract] OR "severe acute respiratory syndrome coronavirus 2 infection"[Title/Abstract] OR "covid 19 virus disease"[Title/Abstract] OR "covid 19 virus disease"[Title/Abstract] OR (("COVID-19"[All Fields] OR "COVID-19"[MeSH Terms] OR "covid 19 vaccines"[All Fields] OR "covid 19 vaccines"[MeSH Terms] OR "covid 19 serotherapy"[All Fields] OR "covid 19 nucleic acid testing"[All Fields] OR "covid 19 nucleic acid testing"[MeSH Terms] OR "covid 19 serological testing"[All Fields] OR "covid 19 serological testing"[MeSH Terms] OR "covid 19 testing"[All Fields] OR "covid 19 testing"[MeSH Terms] OR "SARS-CoV-2"[All Fields] OR "SARS-CoV-2"[MeSH Terms] OR "severe acute respiratory syndrome coronavirus 2"[All Fields] OR "nCoV"[All Fields] OR "2019-nCoV"[All Fields] OR (("Coronavirus"[MeSH Terms] OR "Coronavirus"[All Fields] OR

		"CoV"[All Fields]) AND 2019/11/01:3000/12/31[Date - Publication])) AND "virus diseases"[Title/Abstract]) OR "disease covid 19 virus"[Title/Abstract] OR "virus disease covid 19"[Title/Abstract] OR "sars coronavirus 2 infection"[Title/Abstract] OR "2019 ncov disease"[Title/Abstract] OR "2019 ncov disease"[Title/Abstract] OR "2019 ncov diseases"[Title/Abstract] OR "disease 2019 ncov"[Title/Abstract] OR "covid 19 pandemic"[Title/Abstract] OR "covid 19 pandemic"[Title/Abstract] OR "pandemic covid 19"[Title/Abstract] OR "covid 19 pandemics"[Title/Abstract]	
	5	"gastrointestinal microbiomes"[Title/Abstract] OR "microbiome gastrointestinal"[Title/Abstract] OR "gut microbiome"[Title/Abstract] OR "gut microbiomes"[Title/Abstract] OR "microbiome gut"[Title/Abstract] OR "gut microflora"[Title/Abstract] OR "microflora gut"[Title/Abstract] OR "gut microbiota"[Title/Abstract] OR "gut microbiotas"[Title/Abstract] OR "microbiota gut"[Title/Abstract] OR "gastrointestinal flora"[Title/Abstract] OR "flora gastrointestinal"[Title/Abstract] OR "gut flora"[Title/Abstract] OR "flora gut"[Title/Abstract] OR "gastrointestinal microbiota"[Title/Abstract] OR "gastrointestinal microbiotas"[Title/Abstract] OR "microbiota gastrointestinal"[Title/Abstract] OR "gastrointestinal microbial community"[Title/Abstract] OR "gastrointestinal microbial	91,113

		communities"[Title/Abstract] OR (("Microbiota"[MeSH Terms] OR "Microbiota"[All Fields] OR ("Microbial"[All Fields] AND "Community"[All Fields]) OR "microbial community"[All Fields]) AND "Gastrointestinal"[Title/Abstract]) OR "gastrointestinal microflora"[Title/Abstract] OR "microflora gastrointestinal"[Title/Abstract] OR "gastric microbiome"[Title/Abstract] OR "gastric microbiomes"[Title/Abstract] OR "microbiome gastric"[Title/Abstract] OR "intestinal microbiome"[Title/Abstract] OR "intestinal microbiomes"[Title/Abstract] OR "microbiome intestinal"[Title/Abstract] OR "intestinal microbiota"[Title/Abstract] OR "intestinal microbiotas"[Title/Abstract] OR "microbiota intestinal"[Title/Abstract] OR "intestinal microflora"[Title/Abstract] OR "microflora intestinal"[Title/Abstract] OR "intestinal flora"[Title/Abstract] OR "flora intestinal"[Title/Abstract] OR "enteric bacteria"[Title/Abstract] OR "bacteria enteric"[Title/Abstract]	
	6	"mendelian randomization"[Title/Abstract]	9,211
	7	"COVID-19"[MeSH Terms] OR ("COVID-19"[Title/Abstract] OR "2019 ncov infection"[Title/Abstract] OR "2019 ncov infection"[Title/Abstract] OR "2019 ncov infections"[Title/Abstract] OR "infection 2019 ncov"[Title/Abstract] OR "sars cov 2 infection"[Title/Abstract] OR "infection sars cov 2"[Title/Abstract] OR "sars	409,685

	cov 2 infection"[Title/Abstract] OR "sars cov 2 infections"[Title/Abstract] OR "2019 novel coronavirus disease"[Title/Abstract] OR "2019 novel coronavirus infection"[Title/Abstract] OR "covid 19 virus infection"[Title/Abstract] OR "covid 19 virus infection"[Title/Abstract] OR "covid 19 virus infections"[Title/Abstract] OR "infection covid 19 virus"[Title/Abstract] OR "virus infection covid 19"[Title/Abstract] OR "coronavirus disease 2019"[Title/Abstract] OR "disease 2019 coronavirus"[Title/Abstract] OR "coronavirus disease 19"[Title/Abstract] OR "coronavirus disease 19"[Title/Abstract] OR "severe acute respiratory syndrome coronavirus 2 infection"[Title/Abstract] OR "covid 19 virus disease"[Title/Abstract] OR "covid 19 virus disease"[Title/Abstract] OR (("COVID-19"[All Fields] OR "COVID-19"[MeSH Terms] OR "covid 19 vaccines"[All Fields] OR "covid 19 vaccines"[MeSH Terms] OR "covid 19 serotherapy"[All Fields] OR "covid 19 nucleic acid testing"[All Fields] OR "covid 19 nucleic acid testing"[MeSH Terms] OR "covid 19 serological testing"[All Fields] OR "covid 19 serological testing"[MeSH Terms] OR "covid 19 testing"[All Fields] OR "covid 19 testing"[MeSH Terms] OR "SARS-CoV-2"[All Fields] OR "SARS-CoV-2"[MeSH Terms] OR "severe acute respiratory syndrome coronavirus 2"[All Fields] OR "nCoV"[All Fields] OR "2019-nCoV"[All Fields] OR (("Coronavirus"[MeSH Terms] OR "Coronavirus"[All Fields] OR "CoV"[All Fields]) AND

		2019/11/01:3000/12/31[Date - Publication])) AND "virus diseases"[Title/Abstract]) OR "disease covid 19 virus"[Title/Abstract] OR "virus disease covid 19"[Title/Abstract] OR "sars coronavirus 2 infection"[Title/Abstract] OR "2019 ncov disease"[Title/Abstract] OR "2019 ncov disease"[Title/Abstract] OR "2019 ncov diseases"[Title/Abstract] OR "disease 2019 ncov"[Title/Abstract] OR "covid 19 pandemic"[Title/Abstract] OR "covid 19 pandemic"[Title/Abstract] OR "pandemic covid 19"[Title/Abstract] OR "covid 19 pandemics"[Title/Abstract])	
	8	"Gastrointestinal Microbiome"[MeSH Terms] OR ("gastrointestinal microbiomes"[Title/Abstract] OR "microbiome gastrointestinal"[Title/Abstract] OR "gut microbiome"[Title/Abstract] OR "gut microbiomes"[Title/Abstract] OR "microbiome gut"[Title/Abstract] OR "gut microflora"[Title/Abstract] OR "microflora gut"[Title/Abstract] OR "gut microbiota"[Title/Abstract] OR "gut microbiotas"[Title/Abstract] OR "microbiota gut"[Title/Abstract] OR "gastrointestinal flora"[Title/Abstract] OR "flora gastrointestinal"[Title/Abstract] OR "gut flora"[Title/Abstract] OR "flora gut"[Title/Abstract] OR "gastrointestinal microbiota"[Title/Abstract] OR "gastrointestinal microbiotas"[Title/Abstract] OR "microbiota gastrointestinal"[Title/Abstract] OR "gastrointestinal microbial community"[Title/Abstract] OR "gastrointestinal microbial	98,061

		communities"[Title/Abstract] OR (("Microbiota"[MeSH Terms] OR "Microbiota"[All Fields] OR ("Microbial"[All Fields] AND "Community"[All Fields]) OR "microbial community"[All Fields]) AND "Gastrointestinal"[Title/Abstract]) OR "gastrointestinal microflora"[Title/Abstract] OR "microflora gastrointestinal"[Title/Abstract] OR "gastric microbiome"[Title/Abstract] OR "gastric microbiomes"[Title/Abstract] OR "microbiome gastric"[Title/Abstract] OR "intestinal microbiome"[Title/Abstract] OR "intestinal microbiomes"[Title/Abstract] OR "microbiome intestinal"[Title/Abstract] OR "intestinal microbiota"[Title/Abstract] OR "intestinal microbiotas"[Title/Abstract] OR "microbiota intestinal"[Title/Abstract] OR "intestinal microflora"[Title/Abstract] OR "microflora intestinal"[Title/Abstract] OR "intestinal flora"[Title/Abstract] OR "flora intestinal"[Title/Abstract] OR "enteric bacteria"[Title/Abstract] OR "bacteria enteric"[Title/Abstract])	
	9	"Mendelian Randomization Analysis"[MeSH Terms] OR "mendelian randomization"[Title/Abstract]	9,862
	10	("COVID-19"[MeSH Terms] OR ("COVID-19"[Title/Abstract] OR "2019 ncov infection"[Title/Abstract] OR "2019 ncov infection"[Title/Abstract] OR "2019 ncov infections"[Title/Abstract] OR "infection 2019 ncov"[Title/Abstract] OR "sars cov 2	9

		infection"[Title/Abstract] OR "infection sars cov 2"[Title/Abstract] OR "sars cov 2 infection"[Title/Abstract] OR "sars cov 2 infections"[Title/Abstract] OR "2019 novel coronavirus disease"[Title/Abstract] OR "2019 novel coronavirus infection"[Title/Abstract] OR "covid 19 virus infection"[Title/Abstract] OR "covid 19 virus infection"[Title/Abstract] OR "covid 19 virus infections"[Title/Abstract] OR "infection covid 19 virus"[Title/Abstract] OR "virus infection covid 19"[Title/Abstract] OR "coronavirus disease 2019"[Title/Abstract] OR "disease 2019 coronavirus"[Title/Abstract] OR "coronavirus disease 19"[Title/Abstract] OR "coronavirus disease 19"[Title/Abstract] OR "severe acute respiratory syndrome coronavirus 2 infection"[Title/Abstract] OR "covid 19 virus disease"[Title/Abstract] OR "covid 19 virus disease"[Title/Abstract] OR (("COVID-19"[All Fields] OR "COVID-19"[MeSH Terms] OR "covid 19 vaccines"[All Fields] OR "covid 19 vaccines"[MeSH Terms] OR "covid 19 serotherapy"[All Fields] OR "covid 19 nucleic acid testing"[All Fields] OR "covid 19 nucleic acid testing"[MeSH Terms] OR "covid 19 serological testing"[All Fields] OR "covid 19 serological testing"[MeSH Terms] OR "covid 19 testing"[All Fields] OR "covid 19 testing"[MeSH Terms] OR "SARS-CoV-2"[All Fields] OR "SARS-CoV-2"[MeSH Terms] OR "severe acute respiratory syndrome coronavirus 2"[All Fields] OR "nCoV"[All Fields] OR "2019-nCoV"[All Fields] OR (("Coronavirus"[MeSH Terms] OR

		"Coronavirus"[All Fields] OR "CoV"[All Fields]) AND 2019/11/01:3000/12/31[Date - Publication])) AND "virus diseases"[Title/Abstract]) OR "disease covid 19 virus"[Title/Abstract] OR "virus disease covid 19"[Title/Abstract] OR "sars coronavirus 2 infection"[Title/Abstract] OR "2019 ncov disease"[Title/Abstract] OR "2019 ncov disease"[Title/Abstract] OR "2019 ncov diseases"[Title/Abstract] OR "disease 2019 ncov"[Title/Abstract] OR "covid 19 pandemic"[Title/Abstract] OR "covid 19 pandemic"[Title/Abstract] OR "pandemic covid 19"[Title/Abstract] OR "covid 19 pandemics"[Title/Abstract])) AND ("Gastrointestinal Microbiome"[MeSH Terms] OR ("gastrointestinal microbiomes"[Title/Abstract] OR "microbiome gastrointestinal"[Title/Abstract] OR "gut microbiome"[Title/Abstract] OR "gut microbiomes"[Title/Abstract] OR "microbiome gut"[Title/Abstract] OR "gut microflora"[Title/Abstract] OR "microflora gut"[Title/Abstract] OR "gut microbiota"[Title/Abstract] OR "gut microbiotas"[Title/Abstract] OR "microbiota gut"[Title/Abstract] OR "gastrointestinal flora"[Title/Abstract] OR "flora gastrointestinal"[Title/Abstract] OR "gut flora"[Title/Abstract] OR "flora gut"[Title/Abstract] OR "gastrointestinal microbiota"[Title/Abstract] OR "gastrointestinal microbiotas"[Title/Abstract] OR "microbiota gastrointestinal"[Title/Abstract] OR "gastrointestinal microbial

		community"[Title/Abstract] OR "gastrointestinal microbial communities"[Title/Abstract] OR (("Microbiota"[MeSH Terms] OR "Microbiota"[All Fields] OR ("Microbial"[All Fields] AND "Community"[All Fields]) OR "microbial community"[All Fields]) AND "Gastrointestinal"[Title/Abstract]) OR "gastrointestinal microflora"[Title/Abstract] OR "microflora gastrointestinal"[Title/Abstract] OR "gastric microbiome"[Title/Abstract] OR "gastric microbiomes"[Title/Abstract] OR "microbiome gastric"[Title/Abstract] OR "intestinal microbiome"[Title/Abstract] OR "intestinal microbiomes"[Title/Abstract] OR "microbiome intestinal"[Title/Abstract] OR "intestinal microbiota"[Title/Abstract] OR "intestinal microbiotas"[Title/Abstract] OR "microbiota intestinal"[Title/Abstract] OR "intestinal microflora"[Title/Abstract] OR "microflora intestinal"[Title/Abstract] OR "intestinal flora"[Title/Abstract] OR "flora intestinal"[Title/Abstract] OR "enteric bacteria"[Title/Abstract] OR "bacteria enteric"[Title/Abstract])) AND ("Mendelian Randomization Analysis"[MeSH Terms] OR "mendelian randomization"[Title/Abstract])	
**(c)CochraneLibrarye**	1	MeSH descriptor: [COVID-19] explode all trees	7739
	2	MeSH descriptor: [Gastrointestinal Microbiome] explode all trees	1658
	3	MeSH descriptor: [Mendelian Randomization Analysis] explode all	862

		trees	
	4	("COVID 19"):ti,ab,kw OR ("2019-nCoV Infection"):ti,ab,kw OR ("2019 nCoV Infection"):ti,ab,kw OR ("2019-nCoV Infections"):ti,ab,kw OR ("Infection, 2019-nCoV"):ti,ab,kw OR ("SARS-CoV-2 Infection"):ti,ab,kw OR ("Infection, SARS-CoV-2"):ti,ab,kw OR ("SARS CoV 2 Infection"):ti,ab,kw OR ("SARS-CoV-2 Infections"):ti,ab,kw OR ("2019 Novel Coronavirus Disease"):ti,ab,kw OR ("2019 Novel Coronavirus Infection"):ti,ab,kw OR ("COVID-19 Virus Infection"):ti,ab,kw OR ("COVID 19 Virus Infection"):ti,ab,kw OR ("COVID-19 Virus Infections"):ti,ab,kw OR ("Infection, COVID-19 Virus"):ti,ab,kw OR ("Virus Infection, COVID-19"):ti,ab,kw OR ("Coronavirus Disease 2019"):ti,ab,kw OR ("Disease 2019, Coronavirus"):ti,ab,kw OR ("Coronavirus Disease-19"):ti,ab,kw OR ("Coronavirus Disease 19"):ti,ab,kw OR ("Severe Acute Respiratory Syndrome Coronavirus 2 Infection"):ti,ab,kw OR ("COVID-19 Virus Disease"):ti,ab,kw OR ("COVID 19 Virus Disease"):ti,ab,kw OR ("COVID-19 Virus Diseases"):ti,ab,kw OR ("Disease, COVID-19 Virus"):ti,ab,kw OR ("Virus Disease, COVID-19"):ti,ab,kw OR ("SARS Coronavirus 2 Infection"):ti,ab,kw OR ("2019-nCoV Disease"):ti,ab,kw OR ("2019 nCoV Disease"):ti,ab,kw OR ("2019-nCoV Diseases"):ti,ab,kw OR ("Disease, 2019-nCoV"):ti,ab,kw OR ("COVID-19 Pandemic"):ti,ab,kw OR ("COVID 19 Pandemic"):ti,ab,kw OR ("Pandemic, COVID-19"):ti,ab,kw OR ("COVID-19 Pandemics"):ti,ab,kw	20435
	5	("Gastrointestinal	8662

	Microbiome"):ti,ab,kw OR ("Gastrointestinal Microbiomes"):ti,ab,kw OR ("Microbiome, Gastrointestinal"):ti,ab,kw OR ("Gut Microbiome"):ti,ab,kw OR ("Gut Microbiomes"):ti,ab,kw OR ("Microbiome, Gut"):ti,ab,kw OR ("Gut Microflora"):ti,ab,kw OR ("Microflora, Gut"):ti,ab,kw OR ("Gut Microbiota"):ti,ab,kw OR ("Gut Microbiotas"):ti,ab,kw OR ("Microbiota, Gut"):ti,ab,kw OR ("Gastrointestinal Flora"):ti,ab,kw OR ("Flora, Gastrointestinal"):ti,ab,kw OR ("Gut Flora"):ti,ab,kw OR ("Flora, Gut"):ti,ab,kw OR ("Gastrointestinal Microbiota"):ti,ab,kw OR ("Gastrointestinal Microbiotas"):ti,ab,kw OR ("Microbiota, Gastrointestinal"):ti,ab,kw OR ("Gastrointestinal Microbial Community"):ti,ab,kw OR ("Gastrointestinal Microbial Communities"):ti,ab,kw OR ("Microbial Community, Gastrointestinal"):ti,ab,kw OR ("Gastrointestinal Microflora"):ti,ab,kw OR ("Microflora, Gastrointestinal"):ti,ab,kw OR ("Gastric Microbiome"):ti,ab,kw OR ("Gastric Microbiomes"):ti,ab,kw OR ("Microbiome, Gastric"):ti,ab,kw OR ("Intestinal Microbiome"):ti,ab,kw OR ("Intestinal Microbiomes"):ti,ab,kw OR ("Microbiome, Intestinal"):ti,ab,kw OR ("Intestinal Microbiota"):ti,ab,kw OR ("Intestinal Microbiotas"):ti,ab,kw OR ("Microbiota, Intestinal"):ti,ab,kw OR ("Intestinal Microflora"):ti,ab,kw OR ("Microflora, Intestinal"):ti,ab,kw OR ("Intestinal Flora"):ti,ab,kw OR ("Flora, Intestinal"):ti,ab,kw OR

		("Enteric Bacteria"):ti,ab,kw OR ("Bacteria, Enteric"):ti,ab,kw	
	6	(Mendelian randomization):ti,ab,kw	1711
	7	#1 or #4	20435
	8	#2 or #5	8662
	9	#3 or #6	1711
	10	#7 and #8 and #9	0
**(d) Web of science**	1	coronavirus disease 2019(Topic) OR COVID-19(Topic) OR covid 19(Topic) OR covid 19(Topic) OR 2019-ncov infection(Topic) OR 2019-ncov infection(Topic) OR 2019 ncov infection(Topic) OR 2019 ncov infection(Topic) OR 2019-ncov infections(Topic) OR infection, 2019-ncov(Topic) OR sars-cov-2 infection(Topic) OR sars-cov-2 infection(Topic) OR infection, sars-cov-2(Topic) OR sars cov 2 infection(Topic) OR sars cov 2 infection(Topic) OR sars-cov-2 infections(Topic) OR 2019 novel coronavirus disease(Topic) OR 2019 novel coronavirus disease(Topic) OR 2019 novel coronavirus infection(Topic) OR 2019 novel coronavirus infection(Topic) OR covid-19 virus infection(Topic) OR covid 19 virus infection(Topic) OR covid-19 virus infections(Topic) OR infection, covid-19 virus(Topic) OR virus infection, covid-19(Topic) OR covid19(Topic) OR covid19(Topic) OR coronavirus disease 2019(Topic) OR coronavirus disease 2019(Topic) OR disease 2019, coronavirus(Topic) OR coronavirus disease-19(Topic) OR coronavirus disease-19(Topic) OR coronavirus disease 19(Topic) OR coronavirus disease 19(Topic) OR severe acute respiratory syndrome coronavirus 2 infection(Topic) OR severe acute respiratory syndrome coronavirus 2 infection(Topic) OR	539373

		covid-19 virus disease(Topic) OR covid 19 virus disease(Topic) OR covid-19 virus diseases(Topic) OR disease, covid-19 virus(Topic) OR virus disease, covid-19(Topic) OR sars coronavirus 2 infection(Topic) OR sars coronavirus 2 infection(Topic) OR 2019-ncov disease(Topic) OR 2019-ncov disease(Topic) OR 2019 ncov disease(Topic) OR 2019 ncov disease(Topic) OR 2019-ncov diseases(Topic) OR disease, 2019-ncov(Topic) OR covid-19 pandemic(Topic) OR covid 19 pandemic(Topic) OR pandemic, covid-19(Topic) OR covid-19 pandemics(Topic)	
	2	intestine flora(Topic) OR Gastrointestinal Microbiome(Topic) OR Gastrointestinal Microbiomes(Topic) OR Microbiome, Gastrointestinal(Topic) OR Gut Microbiome(Topic) OR Gut Microbiomes(Topic) OR Microbiome, Gut(Topic) OR Gut Microflora(Topic) OR Microflora, Gut(Topic) OR Gut Microbiota(Topic) OR Gut Microbiotas(Topic) OR Microbiota, Gut(Topic) OR Gastrointestinal Flora(Topic) OR Flora, Gastrointestinal(Topic) OR Gut Flora(Topic) OR Flora, Gut(Topic) OR Gastrointestinal Microbiota(Topic) OR Gastrointestinal Microbiotas(Topic) OR Microbiota, Gastrointestinal(Topic) OR Gastrointestinal Microbial Community(Topic) OR Gastrointestinal Microbial Communities(Topic) OR Microbial Community, Gastrointestinal(Topic) OR Gastrointestinal Microflora(Topic) OR Microflora, Gastrointestinal(Topic) OR Gastric Microbiome(Topic) OR Gastric Microbiomes(Topic) OR Microbiome,	144279

		Gastric(Topic) OR Intestinal Microbiome(Topic) OR Intestinal Microbiomes(Topic) OR Microbiome, Intestinal(Topic) OR Intestinal Microbiota(Topic) OR Intestinal Microbiotas(Topic) OR Microbiota, Intestinal(Topic) OR Intestinal Microflora(Topic) OR Microflora, Intestinal(Topic) OR Intestinal Flora(Topic) OR Flora, Intestinal(Topic) OR Enteric Bacteria(Topic) OR Bacteria, Enteric(Topic)	
	3	mendelian randomization analysis(Topic) OR mendelian randomization(Topic)	12965
	4	#3 AND #2 AND #1	11
**(e)Scopus**	1	TITLE-ABS-KEY ("coronavirus disease" OR "covid-19" OR "covid 19" OR "covid 19" OR "2019-ncov infection" OR "2019-ncov infection" OR "2019 ncov infection" OR "2019 ncov infection" OR "2019-ncov infections" OR "infection, 2019-ncov" OR "sars-cov-2 infection" OR "sars-cov-2 infection" OR "infection, sars-cov-2" OR "sars cov 2 infection" OR "sars cov 2 infection" OR "sars-cov-2 infections" OR "2019 novel coronavirus disease" OR "2019 novel coronavirus disease" OR "2019 novel coronavirus infection" OR "2019 novel coronavirus infection" OR "covid-19 virus infection" OR "covid 19 virus infection" OR "covid-19 virus infections" OR "infection, covid-19 virus" OR "virus infection, covid-19" OR "covid19" OR "covid19" OR "coronavirus disease 2019" OR "coronavirus disease 2019" OR "disease 2019, coronavirus" OR "coronavirus disease-19" OR "coronavirus disease-19" OR "coronavirus disease 19" OR "coronavirus disease 19" OR	640934

		"severe acute respiratory syndrome coronavirus 2 infection" OR "severe acute respiratory syndrome coronavirus 2 infection" OR "covid-19 virus disease" OR "covid 19 virus disease" OR "covid-19 virus diseases" OR "disease, covid-19 virus" OR "virus disease, covid-19" OR "sars coronavirus 2 infection" OR "sars coronavirus 2 infection" OR "2019-ncov disease" OR "2019-ncov disease" OR "2019 ncov disease" OR "2019 ncov disease" OR "2019-ncov diseases" OR "disease, 2019-ncov" OR "covid-19 pandemic" OR "covid 19 pandemic" OR "pandemic, covid-19" OR "covid-19 pandemics")	
	2	TITLE-ABS-KEY ("intestine flora" OR "gastrointestinal microbiome" OR "gastrointestinal microbiomes" OR "microbiome, gastrointestinal" OR "gut microbiome" OR "gut microbiomes" OR "microbiome, gut" OR "gut microflora" OR "microflora, gut" OR "gut microbiota" OR "gut microbiotas" OR "microbiota, gut" OR "gastrointestinal flora" OR "flora, gastrointestinal" OR "gut flora" OR "flora, gut" OR "gastrointestinal microbiota" OR "gastrointestinal microbiotas" OR "microbiota, gastrointestinal" OR "gastrointestinal microbial community" OR "gastrointestinal microbial communities" OR "microbial community, gastrointestinal" OR "gastrointestinal microflora" OR "microflora, gastrointestinal" OR "gastric microbiome" OR "gastric microbiomes" OR "microbiome, gastric" OR "intestinal microbiome" OR "intestinal microbiomes" OR "microbiome, intestinal" OR "intestinal microbiota" OR "intestinal microbiotas"	139934

		OR "microbiota, intestinal" OR "intestinal microflora" OR "microflora, intestinal" OR "intestinal flora" OR "flora, intestinal" OR "enteric bacteria" OR "bacteria, enteric")	
	3	TITLE-ABS-KEY ("mendelian randomization analysis" OR "mendelian randomization")	11199
	4	(TITLE-ABS-KEY("mendelian randomization analysis" OR "mendelian randomization")) AND (TITLE-ABS-KEY("intestine flora" OR "Gastrointestinal Microbiome" OR "Gastrointestinal Microbiomes" OR "Microbiome, Gastrointestinal" OR "Gut Microbiome" OR "Gut Microbiomes" OR "Microbiome, Gut" OR "Gut Microflora" OR "Microflora, Gut" OR "Gut Microbiota" OR "Gut Microbiotas" OR "Microbiota, Gut" OR "Gastrointestinal Flora" OR "Flora, Gastrointestinal" OR "Gut Flora" OR "Flora, Gut" OR "Gastrointestinal Microbiota" OR "Gastrointestinal Microbiotas" OR "Microbiota, Gastrointestinal" OR "Gastrointestinal Microbial Community" OR "Gastrointestinal Microbial Communities" OR "Microbial Community, Gastrointestinal" OR "Gastrointestinal Microflora" OR "Microflora, Gastrointestinal" OR "Gastric Microbiome" OR "Gastric Microbiomes" OR "Microbiome, Gastric" OR "Intestinal Microbiome" OR "Intestinal Microbiomes" OR "Microbiome, Intestinal" OR "Intestinal Microbiota" OR "Intestinal Microbiotas" OR "Microbiota, Intestinal" OR "Intestinal Microflora" OR "Microflora, Intestinal" OR "Intestinal Flora" OR "Flora, Intestinal" OR "Enteric Bacteria" OR "Bacteria, Enteric")) AND	16

		(TITLE-ABS-KEY("coronavirus disease" OR "COVID-19" OR "covid 19" OR "covid 19" OR "2019-ncov infection" OR "2019-ncov infection" OR "2019 ncov infection" OR "2019 ncov infection" OR "2019-ncov infections" OR "infection, 2019-ncov" OR "sars-cov-2 infection" OR "sars-cov-2 infection" OR "infection, sars-cov-2" OR "sars cov 2 infection" OR "sars cov 2 infection" OR "sars-cov-2 infections" OR "2019 novel coronavirus disease" OR "2019 novel coronavirus disease" OR "2019 novel coronavirus infection" OR "2019 novel coronavirus infection" OR "covid-19 virus infection" OR "covid 19 virus infection" OR "covid-19 virus infections" OR "infection, covid-19 virus" OR "virus infection, covid-19" OR "covid19" OR "covid19" OR "coronavirus disease 2019" OR "coronavirus disease 2019" OR "disease 2019, coronavirus" OR "coronavirus disease-19" OR "coronavirus disease-19" OR "coronavirus disease 19" OR "coronavirus disease 19" OR "severe acute respiratory syndrome coronavirus 2 infection" OR "severe acute respiratory syndrome coronavirus 2 infection" OR "covid-19 virus disease" OR "covid 19 virus disease" OR "covid-19 virus diseases" OR "disease, covid-19 virus" OR "virus disease, covid-19" OR "sars coronavirus 2 infection" OR "sars coronavirus 2 infection" OR "2019-ncov disease" OR "2019-ncov disease" OR "2019 ncov disease" OR "2019 ncov disease" OR "2019-ncov diseases" OR "disease, 2019-ncov" OR "covid-19 pandemic" OR "covid 19 pandemic" OR "pandemic, covid-19" OR "covid-19 pandemics"))

Supplementary Table S2 Summary table of the impact of different microflora on new crown pneumonia

Bacterial flora/Metabolites	Label	Disease	SNP Quantities	OR (95%CI) IVW Results
**Phylum**				
**phylum Cyanobacteria**	Weifeng Shang 2023[20]	COVID-19 severity	8	0.85(0.76–0.96)
	Meng-Mei Zhong 2023[21]	COVID-19 severity	8	0.852(0.760–0.955)
**phylum Actinobacteria.id.400**	Yingjian Liu 2024[17]	BP(bacterial pneumonia)	16	2.09(1.13,3.88)
	Jukun Song 2023[25]	COVID-19 hospitalization	20	1.121487(1.028754-1.22258)
		COVID-19 severity	17	1.202516(1.015075-1.42457)
**phylum Lentisphaerae**	Zengbin Li 2023[22]	COVID-19 infection	9	0.93(0.87–0.99)
	Jukun Song 2023[25]	COVID-19 susceptibility	15	1.021896(1.00006-1.044209)
**Class**				
**class Negativicutes**	Weifeng Shang 2023[20]	COVID-19 susceptibility	12	1.05(1.01–1.10)
	Meng-Mei Zhong 2023[21]	COVID-19 susceptibility	13	1.054(1.005–1.105)
	Zengbin Li 2023[22]	COVID-19 infection	12	1.13(1.02–1.26)
	Jukun Song 2023[25]	COVID-19 susceptibility	11	1.069307(1.016779-1.124549)
		COVID-19 hospitalization	11	1.234846(1.112774-1.37031)
		COVID-19 severity	8	1.292966(1.075591-1.554273)
**class Gammaproteobacteria**	Weifeng Shang 2023[20]	COVID-19 susceptibility	7	0.94(0.89–0.99)
	Meng-Mei Zhong 2023[21]	COVID-19 susceptibility	6	0.933(0.879–0.991)
	Jukun Song 2023[25]	COVID-19 susceptibility	10	0.943826(0.898701-0.991217)
**class Actinobacteria**	Han Chen 2023[23]	COVID-19 susceptibility	21	1.156(1.062–1.258)
	Jukun Song 2023[25]	COVID-19 hospitalization	21	1.104484(1.031225-1.182947)

	Hanyu Zhang 2023[26]	COVID-19 hospitalization	1	1.57493389505008(0.504-4.92)
		COVID-19 severity	1	2.53280022758574(1.228-5.224)
**Order**				
**order Gastranaerophilales**	Yingjian Liu 2024[17]	BP(bacterial pneumonia)	9	1.67(1.02,2.74)
	Hanyu Zhang 2023[26]	COVID-19 infection	1	1.19602074416788(0.999-1.432)
		COVID-19 hospitalization	1	1.14110831926724(0.47-2.773)
		COVID-19 severity	1	0.7795799733847(0.45-1.35)
**order Selenomonadales**	Weifeng Shang 2023[20]	COVID-19 susceptibility	12	1.05(1.01–1.10)
	Meng-Mei Zhong 2023[21]	COVID-19 susceptibility	12	1.054(1.005–1.105)
	Zengbin Li 2023[22]	COVID-19 infection	12	1.13(1.02–1.26)
	Jukun Song 2023[25]	COVID-19 susceptibility	15	1.053979(1.010557-1.099266)
		COVID-19 hospitalization	15	1.134561(1.026603-1.253872)
		COVID-19 severity	12	1.188812(1.01203-1.396475)
**order Lactobacillales**	Weifeng Shang 2023[20]	COVID-19 severity	15	0.87(0.76–0.98)
	Meng-Mei Zhong 2023[21]	COVID-19 severity	15	0.867(0.764–0.983)
**order MollicutesRF9**	Weifeng Shang 2023[20]	COVID-19 severity	13	1.14(1.01–1.29)
	Meng-Mei Zhong 2023[21]	COVID-19 severity	13	1.141(1.009–1.291)
	Jukun Song 2023[25]	COVID-19 hospitalization	12	1.126898(1.044468-1.215834)
		COVID-19 severity	15	1.168451(1.017332-1.342018)
**order Bifidobacteriales**	Hanyu Zhang 2023[26]	COVID-19 hospitalization	1	0.648560491804976(0.219-1.917)
		COVID-19 severity	2	0.471(0.286-0.774)
**Family**				
**family Victivallaceae**	Yuxin Zou 2024[19]	COVID-19 severity	12	0.888（0.801-0.984)

	Zengbin Li 2023[22]	COVID-19 hospitalization	12	1.11(1.00–1.24)
**family Streptococcaceae**	Weifeng Shang 2023[20]	COVID-19 susceptibility	14	0.95(0.92–1.00)
	Meng-Mei Zhong 2023[21]	COVID-19 susceptibility	14	0.955(0.916–0.995)
	Hanyu Zhang 2023[26]	COVID-19 infection	1	1.12075212488415(0.837-1.501)
		COVID-19 severity	1	0.999972600375377(0.429-2.332)
**family Streptococcaceae**	Weifeng Shang 2023[20]	COVID-19 susceptibility	14	0.95(0.92–1.00)
	Meng-Mei Zhong 2023[21]	COVID-19 susceptibility	14	0.955(0.916–0.995)
	Hanyu Zhang 2023[26]	COVID-19 infection	1	1.12075212488415(0.837-1.501)
		COVID-19 severity	1	0.999972600375377(0.429-2.332)
**family Bacteroidaceae**	Weifeng Shang 2023[20]	COVID-19 susceptibility	9	1.06(1.01–1.12)
	Meng-Mei Zhong 2023[21]	COVID-19 susceptibility	9	1.06(1.007–1.125)
	Jukun Song 2023[25]	COVID-19 susceptibility	8	1.072539(1.011616-1.137131)
**family Christensenellaceae**	Meng-Mei Zhong 2023[21]	COVID-19 severity	11	0.87(0.77–0.99)
	Jukun Song 2023[25]	COVID-19 hospitalization	13	0.918613(0.846107-0.997332)
**family unknownfamily.id.1000005471**	Jukun Song 2023[25]	COVID-19 hospitalization	12	1.11355(1.026157-1.208386)
		COVID-19 severity	11	1.23367(1.047721-1.452621)
**family Oxalobacteraceae**	Hanyu Zhang 2023[26]	COVID-19 infection	1	0.973361241524337(0.818-1.159)
		COVID-19 hospitalization	1	1.68539507129741(0.716-3.969)
		COVID-19 severity	1	1.23244499853025(0.755-2.012)
**family Peptostreptococcaceae**	Hanyu Zhang 2023[26]	COVID-19 infection	1	1.00904062177387(0.767-1.328)
		COVID-19 severity	1	1.79858455998767(0.824-3.924)
**family Bifidobacteriaceae**	Yingjian Liu 2024[17]	Pneumonia	13	0.68(0.54,0.85)

	Yingjian Liu 2024[17]	BP(bacterial pneumonia)	15	2.05(1.17,3.58)
	Hanyu Zhang 2023[26]	COVID-19 infection	1	1.06235820628227(0.875-1.29)
		COVID-19 hospitalization	1	1.54120056443399(0.521-4.56)
		COVID-19 severity	2	2.124(1.152-3.915)
**family FamilyXI.id.1936**	Yingjian Liu 2024[17]	Pneumonia	8	1.18(1.01,1.38)
	Jukun Song 2023[25]	COVID-19 hospitalization	12	0.95861(0.919248-0.999658)
**Genus**				
**genus Anaerofilum.id.2053**	Yingjian Liu 2024[17]	Pneumonia	7	1.34(1.04,1.72)
	Jukun Song 2023[25]	COVID-19 hospitalization	12	0.945835(0.896604-0.997769)
**genus Marvinbryantia**	Yingjian Liu 2024[17]	BLA(bronchopneumonia、lung abscess)	9	0.41(0.17,1)
	Meng-Mei Zhong 2023[21]	COVID-19 hospitalization	10	0.886(0.812–0.967)
**genus RuminococcaceaeUCG011**	Weifeng Shang 2023[20]	COVID-19 severity	8	0.91(0.83–0.99)
	Jukun Song 2023[25]	COVID-19 severity	8	0.906709(0.832425-0.987621)
	Meng-Mei Zhong 2023[21]	COVID-19 severity		0.907(0.832–0.988)
**genus Tyzzerella3**	Weifeng Shang 2023[20]	COVID-19 severity	13	0.89(0.81–0.97)
	Meng-Mei Zhong 2023[21]	COVID-19 severity	13	0.885(0.810–0.967)
	Jukun Song 2023[25]	COVID-19 hospitalization	18	0.951996(0.910977-0.994862)
		COVID-19 severity	14	0.896826(0.82266-0.97768)
	Hanyu Zhang 2023[26]	COVID-19 severity	1	2.21142565432121(1.246-3.924)
**genus Subdoligranulum**	Meng-Mei Zhong 2023[21]	COVID-19 severity	11	0.807(0.699–0.932)
	Jukun Song 2023[25]	COVID-19 severity	13	0.855125(0.749524-0.975604)

	Weifeng Shang 2023[20]	COVID-19 severity	11	0.8(0.69–0.92)
**genus Bacteroides**	Weifeng Shang 2023[20]	COVID-19 susceptibility	9	1.06(1.01–1.12)
	Meng-Mei Zhong 2023[21]	COVID-19 susceptibility	9	1.064(1.007–1.125)
	Jukun Song 2023[25]	COVID-19 susceptibility	12	1.059099(1.010079-1.110498)
**genus Parasutterella**	Yingjian Liu 2024[17]	BP(bacterial pneumonia)	12	2.75(1.49,5.08)
	Zengbin Li 2023[22]	COVID-19 infection	14	0.89(0.83–0.97)
		COVID-19 hospitalization	14	0.84(0.72–0.98)
	Han Chen 2023[23]	COVID-19 susceptibility	16	0.902(0.836–0.973)
**genus Bifidobacterium.**	Yingjian Liu 2024[17]	BP(bacterial pneumonia)	16	1.93(1.15,3.22)
	Tian, Siyu 2024[18]	COVID-19 hospitalization	13	1.126(1.021–1.242)
	Jukun Song 2023[25]	COVID-19 susceptibility	21	1.030965(1.000158-1.062721)
	Han Yan 2023[27]	COVID-19 susceptibility	2	2.092(1.149-3.808)
**genus RikenellaceaeRC9**	Tian, Siyu 2024[18]	COVID-19 hospitalization	13	1.081(1.019-1.147)
	Weifeng Shang 2023[20]	COVID-19 severity	11	1.09(1.01–1.17)
	Meng-Mei Zhong 2023[21]	COVID-19 severity	8	1.085(1.009–1.167)
**genus LachnospiraceaeUCG008**	Weifeng Shang 2023[20]	COVID-19 severity	11	1.12(1.00–1.26)
	Jukun Song 2023[25]	COVID-19 severity	12	1.110538(1.000231-1.233009)
**genus Olsenella**	Meng-Mei Zhong 2023[21]	COVID-19 hospitalization	11	0.942(0.897–0.990)
	Jukun Song 2023[25]	COVID-19 hospitalization	13	0.938009(0.896461-0.981482)
**genus Eubacterium coprostanoligenes**	Hanyu Zhang 2023[26]	COVID-19 infection	1	0.839701170368478(0.615-1.146)

**group**		COVID-19 hospitalization	1	0.568684261257267(0.145-2.223)
		COVID-19 severity	1	0.792819896331787(0.277-2.271)
**genus Dorea**	Meng-Mei Zhong 2023[21]	COVID-19 hospitalization	10	1.162(1.055–1.279)
	Jukun Song 2023[25]	COVID-19 susceptibility	14	1.048128(1.006111-1.091899)
**genus Ruminococcaceae UCG014**	Zengbin Li 2023[22]	COVID-19 infection	11	0.88(0.80–0.97)
		COVID-19 hospitalization	11	0.79(0.65–0.97)
	Han Chen 2023[23]	COVID-19 susceptibility	9	0.878(0.777–0.992)
**genus Alloprevotella**	Zengbin Li 2023[22]	COVID-19 hospitalization	5	1.25(1.07–1.45)
		COVID-19 severity	5	1.67(1.32–2.11)
	Han Chen 2023[23]	COVID-19 susceptibility	7	1.088(1.021–1.160)
	Han Yan 2023[27]	COVID-19 severity	4	1.627(1.14-2.323)
**genus Prevotella9**	Zengbin Li 2023[22]	COVID-19 hospitalization	14	1.21(1.04–1.41)
	Jukun Song 2023[25]	COVID-19 severity	19	1.108017(1.015604-1.208839)
**genus Ruminococcus gnavus group**	Zengbin Li 2023[22]	COVID-19 severity	12	0.77(0.62–0.95)
	Han Yan 2023[27]	COVID-19 severity	2	1.703(1.018-2.849)
**genus Oxalobacter**	Zengbin Li 2023[22]	COVID-19 severity	11	0.84(0.71–1.00)
	Han Chen 2023[23]	COVID-19 susceptibility	13	0.842(0.712–0.994)
		COVID-19 severity	13	0.842 (0.712–0.994)
	Hanyu Zhang 2023[26]	COVID-19 infection	1	0.872561011613187(0.736-1.035)
		COVID-19	1	0.706206493883378(

		hospitalization		0.3-1.661)
		COVID-19 severity	1	1.09557314891857(0.606-1.981)
	Han Yan 2023[27]	COVID-19 severity	11	0.842(0.709-1.000)
			4	0.752(0.578-0.98)
**genus Ruminiclostridium6**	Zengbin Li 2023[22]	COVID-19 hospitalization	15	0.80(0.69–0.94)
		COVID-19 severity	16	0.78(0.62–0.98)
	Han Yan 2023[27]	COVID-19 severity	14	0.708(0.544-0.921)
**genus.unknowngenus.id.1000005472**	Jukun Song 2023[25]	COVID-19 hospitalization	15	1.101318(1.028715-1.179046)
		COVID-19 severity	11	1.237166(1.064543-1.437781)
**genus Ruminococcustorques group**	Hanyu Zhang 2023[26]	COVID-19 infection	1	0.537(0.391–0.738)
		COVID-19 severity	1	0.536877354869706(0.391-0.738)
**genus Allisonella**	Hanyu Zhang 2023[26]	COVID-19 infection	1	0.999477974302852(0.879-1.137)
		COVID-19 hospitalization	1	0.903849603717244(0.499-1.636)
		COVID-19 severity	1	0.750932133107426(0.464-1.215)
**genus Erysipelatoclostridium**	Hanyu Zhang 2023[26]	COVID-19 infection	1	0.925967316791888(0.737-1.163)
		COVID-19 severity	1	0.66116800731294(0.298-1.468)
**genus Faecalibacterium**	Hanyu Zhang 2023[26]	COVID-19 infection	1	0.947829609211915(0.69-1.303)
		COVID-19 severity	1	0.545239789689792(0.184-1.614)
**genus Peptococcus**	Hanyu Zhang 2023[26]	COVID-19 infection	1	0.957340664124295(0.805-1.138)
		COVID-19 hospitalization	1	0.675976747986784(0.313-1.459)

		COVID-19 severity	1	0.675101722137951(0.419-1.088)
**genus Romboutsia**	Hanyu Zhang 2023[26]	COVID-19 infection	1	0.991370771376931(0.755-1.302)
		COVID-19 severity	1	1.78794246889422(0.825-3.874)
**genus RuminococcaceaeUCG009**	Hanyu Zhang 2023[26]	COVID-19 infection	1	0.997685345952551(0.774-1.285)
		COVID-19 severity	1	1.09160755405964(0.494-2.414)
**genus Streptococcus**	Hanyu Zhang 2023[26]	COVID-19 infection	1	1.11442825766029(0.844-1.471)
		COVID-19 severity	1	0.999973966838869(0.447-2.235)
**genus Intestinibacter**	Hanyu Zhang 2023[26]	COVID-19 infection	1	1.14628731724782(0.846-1.553)
		COVID-19 hospitalization	1	1.38991028236733(0.301-6.42)
		COVID-19 severity	1	1.57226695768584(0.604-4.094)
**genus Enterorhabdus**	Hanyu Zhang 2023[26]	COVID-19 infection	1	1.1672334778462(0.958-1.422)
		COVID-19 hospitalization	1	1.02698051702283(0.335-3.146)
		COVID-19 severity	1	0.76744596953411(0.447-1.317)
**Gut production of the SCFA butyrate**	Wanqiang Lv 2023[24]	COVID-19 hospitalization	8	0.96832539912073(0.94416699163366-0.993101947950892)
		COVID-19 severity	7	1.00845602976638(0.96356320994773-1.05544042515628)
**Fecal propionate**	Wanqiang Lv 2023[24]	COVID-19 hospitalization	3	0.941469863248343(0.808083479895778-1.09687368379214)
		COVID-19 severity	3	0.968794000309792(0.857951748844421-1.09395640990347)

Supplementary Table S3 Summary of bacterial flora of COVID-19 with different severities

Disease	Label	Bacterial flora/Metabolites	SNP Quantities	OR（95%CI）IVW Results
**COVID-19 susceptibility**	Weifeng Shang 2023[20]	class Negativicutes	12	1.05(1.01–1.10)
		class Gammaproteobacteria	7	0.94(0.89–0.99)
		order Selenomonadales	12	1.05(1.01–1.10)
		family Streptococcaceae	14	0.95(0.92–1.00)
		family Bacteroidaceae	9	1.06(1.01–1.12)
		genus Bacteroides	9	1.06(1.01–1.12)
	Meng-Mei Zhong 2023[21]	class Gammaproteobacteria	6	0.933(0.879–0.991)
		family Streptococcaceae	14	0.955(0.916–0.995)
		class Negativicutes	13	1.054(1.005–1.105)
		order Selenomonadales	12	1.054(1.005–1.105)
		family Bacteroidaceae	9	1.06(1.007–1.125)
		genus Bacteroides	9	1.064(1.007–1.125)
	Jukun Song 2023[25]	class.Gammaproteobacteria.id.3303	10	0.943826(0.898701-0.991217)
		phylum.Lentisphaerae.id.2238	15	1.021896(1.00006-1.044209)
		genus.Eisenbergiella.id.11304	12	1.027563(1.000392-1.055472)
		genus.unknowngenus.id.2041	13	1.03016(1.000408-1.060798)
		genus.Bifidobacterium.id.436	21	1.030965(1.000158-1.062721)
		genus.unknowngenus.id.2001	11	1.039753(1.002465-1.078429)
		genus.Flavonifractor.id.2059	10	1.044181(1.002618-1.087467)
		genus.Dorea.id.1997	14	1.048128(1.006111-1.091899)
		order.Selenomonadales.id.2165	15	1.053979(1.010557-1.099266)

		class.Deltaproteobacteria.id.3087	13	1.055903(1.003375-1.111118)
		genus.Bacteroides.id.918	12	1.059099(1.010079-1.110498)
		class.Negativicutes.id.2164	11	1.069307(1.016779-1.124549)
		family.Bacteroidaceae.id.917	8	1.072539(1.011616-1.137131)
	Han Chen 2023[23]	Genus Butyricimonas	13	0.919(0.847–0.998)
		Genus Parasutterella	16	0.902(0.836–0.973)
		Genus Ruminococcaceae UCG014	9	0.878(0.777–0.992)
		Genus Oxalobacter	13	0.842(0.712–0.994)
		Class Actinobacteria	21	1.156(1.062–1.258)
		Class Alphaproteobacteria	9	1.102(1.004–1.211)
		Genus Alloprevotella	7	1.088(1.021–1.160)
		Genus Coprococcus	10	1.159(1.030–1.304)
		Genus Erysipelatoclostri-dium	13	1.083(1.001–1.172)
**COVID-19 infection**	Zengbin Li 2023[22]	phylum Lentisphaerae	9	0.93(0.87–0.99)
		family Alcaligenaceae	12	0.87(0.78–0.96)
		family Lachnospiraceae	17	0.91(0.84–1.00)
		genus Dialister	11	0.91(0.82–1.00)
		genus Parasutterella	14	0.89(0.83–0.97)
		genus Ruminococcaceae UCG003	12	0.90(0.82–0.99)
		genus Ruminococcaceae UCG014	11	0.88(0.80–0.97)
		class Negativicutes	12	1.13(1.02–1.26)
		order Selenomonadales	12	1.13(1.02–1.26)
		genus Phascolarctobacterium	9	1.13(1.02–1.25)

	Hanyu Zhang 2023[26]	**genus Ruminococcustorquesgroup**	**1**	**0.537(0.391–0.738)**
		**genus Ruminococcaceae UCG013**	**1**	**1.38206616435633(1.025-1.863)**
		**genus Ruminococcus1**	**1**	**0.734645873967539(0.545-0.99)**
		**genus Allisonella**	**1**	**0.999477974302852(0.879-1.137)**
		genus Eubacteriumcoprostanoligenesgroup	1	0.839701170368478(0.615-1.146)
		genus Oxalobacter	1	0.872561011613187(0.736-1.035)
		genus Erysipelatoclostridium	1	0.925967316791888(0.737-1.163)
		genus Faecalibacterium	1	0.947829609211915(0.69-1.303)
		genus Peptococcus	1	0.957340664124295(0.805-1.138)
		family Oxalobacteraceae	1	0.973361241524337(0.818-1.159)
		genus Romboutsia	1	0.991370771376931(0.755-1.302)
		genus RuminococcaceaeUCG009	1	0.997685345952551(0.774-1.285)
		family Peptostreptococcaceae	1	1.00904062177387(0.767-1.328)
		genus Bifidobacteriaceae	1	1.06235820628227(0.875-1.29)
		genus Streptococcus	1	1.11442825766029(0.844-1.471)
		family Streptococcaceae	1	1.12075212488415(0.837-1.501)
		genus Intestinibacter	1	1.14628731724782(0.846-1.553)
		genus Enterorhabdus	1	1.1672334778462(0.958-1.422)
		order Gastranaerophilales	1	1.19602074416788(0.999-1.432)
**COVID-19 hospitalizati**	Tian, Siyu 2024[18]	Bifidobacterium.id.436	13	1.126(1.021–1.242)

**on**		LachnospiraceaeUCG010.id.11330	10	1.139(1.009-1.287)
		RikenellaceaeRC9gutgroup.id.11191	13	1.081(1.019-1.147)
		RuminococcaceaeUCG014.id.11371	11	0.822(0.782-0.995)
	Meng-Mei Zhong 2023[21]	genus Marvinbryantia	10	0.886(0.812–0.967)
		genus Olsenella	11	0.942(0.897–0.990)
		family Veillonellaceae	19	1.069(1.002–1.140)
		genus Eubacteriumruminantiumgroup	18	1.065(1.010–1.123)
		genus Dorea	10	1.162(1.055–1.279)
	Zengbin Li 2023[22]	genus Alistipes	14	0.78(0.63–0.96)
		genus Parasutterella	14	0.84(0.72–0.98)
		genus Ruminiclostridium6	15	0.80(0.69–0.94)
		genus Ruminococcaceae UCG014	11	0.79(0.65–0.97)
		family FamilyXIII	10	1.30(1.03–1.64)
		family Victivallaceae	12	1.11(1.00–1.24)
		genus Alloprevotella	5	1.25(1.07–1.45)
		genus Prevotella9	14	1.21(1.04–1.41)
	Wanqiang Lv 2023[24]	Gut production of the SCFA butyrate	8	0.96832539912073(0.94416699163366-0.993101947950892)
		Fecal propionate	3	0.941469863248343(0.808083479895778-1.09687368379214)
	Jukun Song 2023[25]	family.Christensenellaceae.id.1866	13	0.918613(0.846107-0.997332)
		genus.Eubacteriumoxidoreducensgroup.id.11339	9	0.934016(0.87255-0.999811)
		genus.Olsenella.id.822	13	0.938009(0.896461-0.981482)

		genus.Anaerofilum.id.2053	12	0.945835(0.896604-0.997769)
		genus.Tyzzerella3.id.113.35	18	0.951996(0.910977-0.994862)
		family.FamilyXI.id.1936	12	0.95861(0.919248-0.999658)
		order.Bacteroidales.id.913	16	1.092775(1.01484-1.176694)
		genus.unknowngenus.id.1000005472	15	1.101318(1.028715-1.179046)
		class.Actinobacteria.id.419	21	1.104484(1.031225-1.182947)
		family.unknownfamily.id.1000005471	12	1.11355(1.026157-1.208386)
		phylum.Actinobacteria.id 400	20	1.121487(1.028754-1.22258)
		order.MollicutesRF9.id.11579	12	1.126898(1.044468-1.215834)
		order.Selenomonadales.id.2165	15	1.134561(1.026603-1.253872)
		class.Negativicutes.id.2164	11	1.234846(1.112774-1.37031)
	Hanyu Zhang 2023[26]	Eubacteriumcoprostanoligenesgroup	1	0.568684261257267(0.145-2.223)
		Bifidobacteriales	1	0.648560491804976(0.219-1.917)
		genus Peptococcus	1	0.675976747986784(0.313-1.459)
		Oxalobacter	1	0.706206493883378(0.3-1.661)
		Allisonella	1	0.903849603717244(0.499-1.636)
		Enterorhabdus	1	1.02698051702283(0.335-3.146)
		Gastranaerophilales	1	1.14110831926724(0.47-2.773)
		Intestinibacter	1	1.38991028236733(0.301-6.42)
		Bifidobacteriaceae	1	1.54120056443399(0.521-4.56)
		class Actinobacteria	1	1.57493389505008(0.504-4.92)
		family Oxalobacteraceae	1	1.68539507129741(0.716-3.969)

**COVID-19 severe**	Tian, Siyu 2024[18]	Intestinimas.id.2062	16	1.179(1.006-1.383)
	Yuxin Zou 2024[19]	Victivallaceae	12	0.888（0.801-0.984）
	Weifeng Shang 2023[20]	phylum Cyanobacteria	8	0.85(0.76–0.96)
		order Lactobacillales	15	0.87(0.76–0.98)
		family Christensenellaceae	11	0.87(0.77–0.99)
		genus Subdoligranulum	11	0.8(0.69–0.92)
		genus Tyzzerella3	13	0.89(0.81–0.97)
		genus RuminococcaceaeUCG011	8	0.91(0.83–0.99)
		order MollicutesRF9	13	1.14(1.01–1.29)
		genus RikenellaceaeRC9	11	1.09(1.01–1.17)
		genus LachnospiraceaeUCG008	11	1.12(1.00–1.26)
	Meng-Mei Zhong 2023[21]	phylum Cyanobacteria	8	0.852(0.760–0.955)
		order Lactobacillales	15	0.867(0.764–0.983)
		genus RuminococcaceaeUCG011	11	0.907(0.832–0.988)
		genus Subdoligranulum	11	0.807(0.699–0.932)
		genus Tyzzerella3	13	0.885(0.810–0.967)
		order MollicutesRF9	13	1.141(1.009–1.291)
		genus RikenellaceaeRC9	8	1.085(1.009–1.167)
	Zengbin Li 2023[22]	genus Ruminococcus gnavus group	12	0.77(0.62–0.95)
		genus Oxalobacter	11	0.84(0.71–1.00)
		genus Ruminiclostridium6	16	0.78(0.62–0.98)
		genus Alloprevotella	5	1.67(1.32–2.11)
	Han Chen 2023[23]	Genus Oxalobacter	13	0.842 (0.712–0.994)
	Wanqiang Lv 2023[24]	Gut production of the SCFA butyrate	7	1.00845602976638(0.96356320994773-1.055440425156

				28)
		Fecal propionate	3	0.968794000309792(0.857951748844421-1.09395640990347)
	Jukun Song 2023[25]	genus.Subdoligranulum.id.2070	13	0.855125(0.749524-0.975604)
		genus.Tyzzerella3.id.11335	14	0.896826(0.82266-0.97768)
		genus.RuminococcaceaeUCG011.id.11368	8	0.906709(0.832425-0.987621)
		genus.Prevotella9.id.11183	19	1.108017(1.015604-1.208839)
		genus.LachnospiraceaeUCG008.id.11328	12	1.110538(1.000231-1.233009)
		family.BacteroidalesS24.7group.id.11173	10	1.149522(1.02712-1.28651)
		genus.unknowngenus.id.1000005479	6	1.173132(1.004522-1.370044)
		order.Selenomonadales.id.2165	12	1.188812(1.01203-1.396475)
		phylum.Actinobacteria.id.400	17	1.202516(1.015075-1.42457)
		family.unknownfamily.id.1000005471	11	1.23367(1.047721-1.452621)
		genus.unknowngenus.id.1000005472	11	1.237166(1.064543-1.437781)
		class.Negativicutes.id.2164	8	1.292966(1.075591-1.554273)
		order.MollicutesRF9.id 11579	15	1.168451(1.017332-1.342018)
	Hanyu Zhang 2023[26]	**order Bifidobacteriales**	**2**	**0.471(0.286-0.774)**
		**genus Ruminococcustorquesgroup**	**1**	**0.536877354869706(0.391-0.738)**
		**genus Bifidobacteriaceae**	**2**	**2.124(1.152-3.915)**
		**genus Tyzzerella3**	**1**	**2.21142565432121(1.246-3.924)**
		**class Actinobacteria**	**1**	**2.53280022758574(1.228-5.224)**
		genus Faecalibacterium	1	0.545239789689792(0.184-1.614)

	genus Erysipelatoclostridium	1	0.66116800731294(0.298-1.468)
	genus Peptococcus	1	0.675101722137951(0.419-1.088)
	genus Allisonella	1	0.750932133107426(0.464-1.215)
	genus Enterorhabdus	1	0.76744596953411(0.447-1.317)
	order Gastranaerophilales	1	0.7795799733847(0.45-1.35)
	genus Eubacteriumcoprostanoligenesgroup	1	0.792819896331787(0.277-2.271)
	family Streptococcaceae	1	0.999972600375377(0.429-2.332)
	genus Streptococcus	1	0.999973966838869(0.447-2.235)
	genus RuminococcaceaeUCG009	1	1.09160755405964(0.494-2.414)
	genus Oxalobacter	1	1.09557314891857(0.606-1.981)
	family Oxalobacteraceae	1	1.23244499853025(0.755-2.012)
	genus Intestinibacter	1	1.57226695768584(0.604-4.094)
	genus Romboutsia	1	1.78794246889422(0.825-3.874)
	family Peptostreptococcaceae	1	1.79858455998767(0.824-3.924)
Han Yan 2023[27]	Ruminiclostridium6	14	0.708(0.544-0.921)
	unknowngenus.id.1000001215	5	0.72(0.536-0.966)
	Oxalobacter	4	0.752(0.578-0.98)
	Butyrivibric	14	0.83(0.69-1.000)

		Oxalobacter	11	0.842(0.709-1.000)
		Howardella	7	1.264(1.009-1.583)
		Alloprevotella	4	1.627(1.14-2.323)
		Ruminococcus gnavus group	2	1.703(1.018-2.849)
		Bifidobacterium	2	2.092(1.149-3.808)
**Pneumonia**	Yingjian Liu 2024[17]	genus.Anaerofilum.id.2053	7	1.34(1.04,1.72)
		family.Bifidobacteriaceae.id.433	13	0.68(0.54,0.85)
		family.Coriobacteriaceae.id.811	17	1.34(1,1.81)
		order.Coriobacteriales.id.810	17	1.34(1,1.81)
		class.Coriobacteriia.id.809	17	1.34(1,1.81)
		family.FamilyXI.id.1936	8	1.18(1.01,1.38)
		genus.LachnospiraceaeND3007group.id.11317	3	2.1(1.17,3.78)
**BP(bacterial pneumonia)**	Yingjian Liu 2024[17]	genus.Parasutterella.id.2892	12	2.75(1.49,5.08)
		phylum.Actinobacteria.id.400	16	2.09(1.13,3.88)
		family.Bifidobacteriaceae.id.433	15	2.05(1.17,3.58)
		genus.Bifidobacterium.id.436	16	1.93(1.15,3.22)
		family.Enterobacteriaceae.id.3469	7	3.39(1.35,8.48)
		order.Enterobacteriale	7	3.39(1.35,8.48)

		s.id.3468		
		order.Gastranaerophilales.id.1591	9	1.67(1.02,2.74)
		family.Rhodospirillaceae.id.2717	14	0.55(0.33,0.91)
		order.Rhodospirillales.id.2667	13	0.56(0.33,0.95)
**BLA(bronchopneumonia、lung abscess)**	Yingjian Liu 2024[17]	genus.Odoribacter.id.952	3	0.17(0.03,0.97)
		genus.Paraprevotella.id.962	12	0.54(0.3,0.94)
		phylum.Bacteroidetes.id.905	11	0.32(0.11,0.92)
		genus.ChristensenellaceaeR.7group.id.11283	9	0.25(0.09,0.73)
		genus.Fusicatenibacter.id.11305	18	2.2(1.03,4.7)
		genus.Marvinbryantia.id.2005	9	0.41(0.17,1)
		class.Methanobacteria.id.119	10	1.58(1,2.5)
		family.Methanobacteriaceae.id.121	10	1.58(1,2.5)
		order.Methanobacteriales.id.120	10	1.58(1,2.5)
		genus.Methanobrevibacter.id.123	6	1.91(1.05,3.47)
		family.Peptococcaceae.id.2024	10	2.03(1.01,4.07)
		family.Porphyromonadaceae.id.943	9	4.93(1.2,20.15)
**PP(pneumococcal pneumonia)**	Yingjian Liu 2024[17]	genus.Adlercreutzia.id.812	5	0.74(0.56,0.97)
		genus.Holdemanella.id.11393	10	1.2(1.02,1.41)
		genus.Lachnospira.id.2004	6	0.66(0.47,0.93)
		genus.LachnospiraceaeNC2004group.id.11316	9	0.77(0.65,0.91)
		family.Rikenellaceae.id.967	21	1.31(1.1,1.57)
